# Genome-Wide Screening Identifies Gene AKR1C1 Critical for Resistance to Pirarubicin in Bladder Cancer

**DOI:** 10.3390/cancers15092487

**Published:** 2023-04-26

**Authors:** Zhenyu Nie, Yuanhui Gao, Mei Chen, Yanling Peng, Na Guo, Hui Cao, Denggao Huang, Xin Gao, Shufang Zhang

**Affiliations:** 1 Central Laboratory, Affiliated Haikou Hospital of Xiangya Medical College, Central South University, Haikou 570208, China; nzy0613@mail.csu.edu.cn (Z.N.);; 2 Graduate School of Chinese Academy of Medical Sciences & Peking Union Medical College, Tsinghua University, Beijing 100190, China; 3 The Key Laboratory of Geriatrics, Beijing Institute of Geriatrics, Beijing Hospital, National Center of Gerontology, National Health Commission, Institute of Geriatric Medicine, Chinese Academy of Medical Sciences, Beijing 100730, China

**Keywords:** CRISPR/dCas9-SAM, bladder cancer, pirarubicin, drug resistance, AKR1C1

## Abstract

**Simple Summary:**

Bladder cancer is one of the most common malignant tumors in the urinary system. Pirarubicin (THP) perfusion therapy is an important treatment method for non-muscle-invasive bladder cancer. However, an increasing number of cases of recurrence or progression of bladder cancer caused by THP resistance have been reported. Therefore, we urgently need to find the critical genes that cause THP resistance in bladder cancer. In this study, we obtained the critical gene AKR1C1 that causes resistance of bladder cancer to THP via CRISPR/dCas9 SAM. This study not only verified that AKR1C1 can cause resistance of bladder cancer cells to THP both in in vivo and in vitro, but also found that THP treatment can gradually increase the expression of AKR1C1, thus causing resistance to the drug. In addition, we also found that aspirin, the AKR1C1 inhibitor, and tempol, the ROS scavenger, can effectively overcome the drug resistance caused by AKR1C1.

**Abstract:**

Non-muscle-invasive bladder cancer (NMIBC) is a common tumor of the urinary system. Given its high rates of recurrence, progression, and drug resistance, NMIBC seriously affects the quality of life and limits the survival time of patients. Pirarubicin (THP) is a bladder infusion chemotherapy drug recommended by the guidelines for NMIBC. Although the widespread use of THP reduces the recurrence rate of NMIBC, 10–50% of patients still suffer from tumor recurrence, which is closely related to tumor resistance to chemotherapy drugs. This study was performed to screen the critical genes causing THP resistance in bladder cancer cell lines by using the CRISPR/dCas9-SAM system. Thus, AKR1C1 was screened. Results showed that the high expression of AKR1C1 could enhance the drug resistance of bladder cancer to THP both in vivo and in vitro. This gene could reduce the levels of 4-hydroxynonenal and reactive oxygen species (ROS) and resist THP-induced apoptosis. However, AKR1C1 did not affect the proliferation, invasion, or migration of the bladder cancer cells. Aspirin, which is an AKR1C1 inhibitor, could help reduce the drug resistance caused by AKR1C1. After receiving THP treatment, the bladder cancer cell lines could upregulate the expression of the AKR1C1 gene through the ROS/KEAP1/NRF2 pathway, leading to resistance to THP treatment. Using tempol, which is an inhibitor of ROS, could prevent the upregulation of AKR1C1 expression.

## 1. Introduction

Bladder cancer is one of the most common malignant tumors in the urinary system [1]. According to cancer statistics, in 2023, the incidence rate of bladder cancer in the United States is estimated to be 82,290 cases, with an expected mortality rate of 16,170 cases [2]. In China, cancer statistics show that 91,893 new cases and 42,973 deaths due to bladder cancer were recorded in 2020 [3]. Bladder cancer is a very broad concept that ranges from low-risk non-muscle invasive bladder cancer (NMIBC) to high-risk muscle-invasive bladder cancer (MIBC). The most important problem for patients with low-risk and moderate-risk NMIBC is the high recurrence rate and five-year relapse-free survival rates of only 43% and 33%, respectively [4]. By contrast, the main problem faced by patients with MIBC is the 50–70% tumor metastasis rate. The total five-year survival rate of patients with advanced MIBC is only 4.8% because of the extremely high metastasis rate [5]. NMIBC is generally believed to be caused by hyperplasia of the urinary tract epithelium, with the loss of chromosome 9 at an early stage. Meanwhile, MIBC is known to be due to abnormal cell development and closely related to genetic instability [6]. Although the two types of bladder cancer have noted differences in genetics and molecular biology, 10–15% of NMIBC still progress to MIBC [7]. For NMIBC patients, the prognosis of MIBC is poor, and the five-year survival rate is extremely low. Therefore, the residual tumor cells treated after tumor resection have a significant impact on the quality of life, health, and economy of patients.

**Figure 1:**
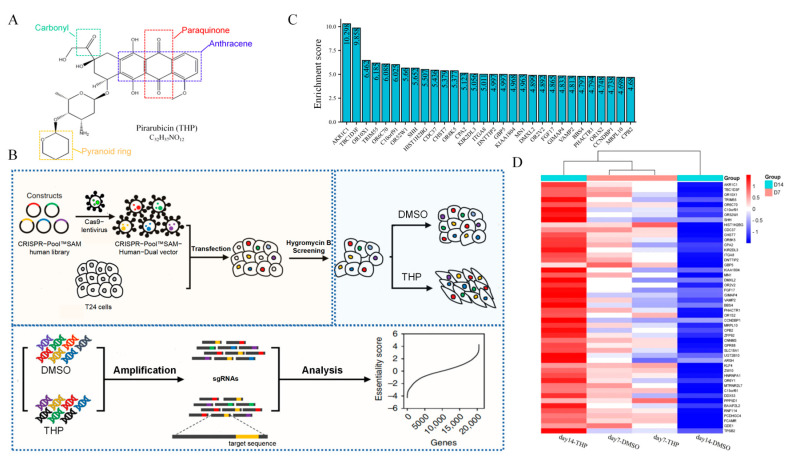
(**A**) The chemical structure of THP and its important functional groups. (**B**) Crispr/dCas9 SAM screening flow diagram. (**C**) Top 30 genes and their Enrichment score. (**D**) The expression of the top 50 genes in the four groups.

Pirarubicin (THP; Figure 1A) is a bladder perfusion drug recommended by the guidelines of NMIBC and commonly used in clinical practice, with a usual dose of 30–50 mg. THP is a new generation of synthetic anthracycline anti-tumor drugs. It plays an antitumor role by inhibiting DNA polymerase to interfere with the synthesis of DNA in cells, causing cell cycle arrest, and promoting tumor cell apoptosis [8]. The chemical structure of THP has another pyran ring on the basis of doxorubicin. This structural change not only enhances the anticancer activity of THP but also increases its distribution concentration in tumor cells and shortens its half-life to reduce its lethality to normal cells [9]. Although the widespread use of THP significantly reduces the recurrence rate of NMIBC, 10–50% of patients still have tumor recurrence within 5 years after receiving THP infusion chemotherapy [10].

The CRISPR/dCas9-SAM system is an improved technology based on CRISPR/Cas9 [11]. The system fuses the nuclease-head mutants of Cas9 (dCas9) without endonuclease activity with the transcription activator VP64. Under the guidance of sgRNA, dCas9-VP64 fusion protein combines with the specific promoter of the genome and collects the transcription activation complex MS2-p63-HSF1. Thus, the transcription of endogenous genes is activated, realizing the expression and activation of specific genes. The system is named the CRISPR/dCas9-SAM system [12], because it can recruit additional transcription activating factors as a synergistic activation mediator (SAM). Compared with the previous CRISPR/Cas9 system, which knocks out target genes as the mechanism, CRISPR/dCas9-SAM involves the overexpression of endogenous target genes. Therefore, the latter technology is widely used in the exploration of drug resistance genes in pathogenic microorganisms [13].

The aldo-keto reductase family 1 member C1 (AKR1C1) is a member of the aldo-keto reductase (AKR) superfamily [14]. This molecule can use nicotinamide adenine dinucleotide phosphate (NADPH) as a coenzyme to reduce carbonyl groups in aldehydes and ketones to hydroxyl groups, thus generating corresponding alcohols [15]. AKRs are widely distributed in prokaryotes and eukaryotes, with 16 subfamilies and more than 190 family members [16]. The AKR1C1 protein coding gene is located on chromosome 10.p15–p14 and consists of 12 exons [16]. The AKR1C1 protein is composed of 323 amino acids with a relative molecular weight of 36 kDa [16]. AKR1C1 participates in various normal physiological and biochemical reactions, and its highest mRNA expression level is observed in the lungs, followed by the liver, testes, breasts, endometrium, and brain [17]. This protein is involved in the reduction of progesterone to 20-hydroxyprogesterone through NADPH catalysis and the reduction of progesterone concentration, which is closely related to premature delivery, endometriosis, and endometrial cancer. In addition, AKR1C1 is involved in the metabolism of multiple drugs. Different drugs, especially antitumor ones, can increase the reactive oxygen species (ROS) in cells, thus causing cell dysfunction or apoptosis through different signaling mechanisms. AKR1C1 can regulate drug metabolism by reducing the production of ROS, clearing oxygen free radicals, and inactivating the active components of various anticancer drugs [17]. AKR1C1 also participates in the metabolism of chemical carcinogens, such as polycyclic aromatic hydrocarbons. AKR1C1 can participate in the metabolism of polycyclic aromatic hydrocarbons and reduce them to corresponding o-quinones with carcinogenic activity [18]. AKR1C1 has been reported to be significantly overexpressed in the metastatic tissues of bladder cancer. Knocking down AKR1C1 in metastatic tissues can inhibit its invasion ability [19]. However, in primary bladder cancer, the drug resistance mechanism of AKR1C1 and THP has not yet been elucidated.

In this study, the key genes of THP resistance in the T24 human bladder cancer cell line were screened through the CRISPR/dCas9-SAM genome-wide library. The results showed that AKR1C1 was the critical gene that caused THP resistance both in vivo and in vitro. The effect and mechanism of AKR1C1 on THP resistance in bladder cancer were explored using cell biology, molecular biology, and pharmacology and combined with AKR1C1 inhibitor (aspirin) and ROS inhibitor (scavenger) to rescue drug-resistant bladder cancer cells to recover their sensitivity to THP. The results provide a new theoretical basis for the exploration of the mechanism of THP resistance in bladder cancer. In addition, a novel theoretical foundation and therapeutic target for the prognostic classification and individualized treatment of bladder cancer have been established.

## 2. Materials and Methods

### 2.1. Crispr Library, Lentivirus, Plasmid, and Cell Lines

Library: CRISPR-Pool™ SAM human library was purchased from Shanghai Genechem Gene Chemical Co., Ltd. (Shanghai, China). The library contained 70,290 SAM-sgRNA targets and 23,430 transcripts of coding genes.

Lentivirus: CRISPR-Pool™ SAM-Human-Dual vector lentivirus was purchased from Shanghai Genechem. The lentivirus system was a two-vector system, which contained two different plasmids.

Tool plasmid: The lentivirus packaging process involved two auxiliary plasmids, namely, lentivirus packaging auxiliary plasmid 1.0 (pHelper 1.0) and lentivirus packaging auxiliary plasmid 2.0 (pHelper 2.0). The plasmids were purchased from Shanghai Genechem Gene Chemical Co., Ltd.

Cell lines: The cell line packed with lentivirus was HEK293T cell, which was an adherent-dependent epithelioid cell, and was cultured in DMEM containing 10% fetal bovine serum (FBS). The selected target cell line was T24 bladder cancer cell line, which was cultured in Roswell Park Memorial Institute (RPMI) 1640 medium with 10% FBS. The RT4 cell line was cultured in McCoy’s 5A medium with 10% FBS. All cells were maintained in a 37 ℃ cell incubator with 5% CO_2_. The cells were resuscitated and subcultured twice before subsequent experiments. All cell lines were purchased from the cell bank of the Typical Culture Preservation Committee of the Chinese Academy of Sciences.

### 2.2. Clinical Tissues, Tissue Microarray, and Animals

The clinical tissues were from patients with bladder cancer who underwent surgery in the Affiliated Haikou Hospital of Xiangya Medical College, Central South University (Table 1 lists the information of patients). After informed consent was signed by the patient and the patient’s family, the removed primary bladder cancer tissues and tumor tissue from recurrent bladder cancer were stored at the bladder cancer clinical sample bank. None of the patients with bladder cancer had other malignant tumors and underwent a biopsy. The pathological diagnosis was bladder cancer. None of the patients had received neoadjuvant radiotherapy, chemotherapy, or other special treatments before the first surgery. The tissue microarray of bladder cancer and normal tissues surrounding the tumor were purchased from Bioaitech Biotechnology Co., Ltd. (Xi’an, China, U812101). The microarray contained 23 cases of noninvasive urothelial carcinoma, 43 cases of invasive urothelial carcinoma, 4 cases of urothelial carcinoma with squamous tissue, and 11 cases of paraneoplastic tissue, of which 7 cases were paraneoplastic and tumor-matched (Table 2). In this study, BALB/c nude mice (4–6 weeks old) were purchased from the Hunan SJA Laboratory Animal Co., Ltd. (Changsha, China). All animal care and related experiments outlined in this study were performed in accordance with the Guidelines for the Care and Use of Laboratory Animals drafted by the US National Institutes of Health (NIH Publication, 8th Edition. 2011). The Institutional Animal Care and Use Committees of Xiangya Medical College and Central Southern University approved the animal protocols used in this study. Adult 4- to 6-week-old BALB/c mice weighing 20–22 g were housed under pathogen-free conditions in a temperature-controlled environment at 22–24 °C, humidity-controlled environment at 40–70% under a 12 h light/dark cycle with ad libitum access to water and food. Male and female mice were raised separately, with 3–5 mice per cage. If there was any mouse fighting behavior, these mice were divided into cages again. Mice were generally in good health before transplanting tumor cells.

Recipe for THP in vivo injection: THP storage solution was made from 1.2 mg of THP dissolved in 150 μL DMSO. Then, every 150 μL storage solution was mixed with 450 μL PEG3000, and then added to 300 μL Tween 80. Finally, the mixed compound contained 2100 μL ddH_2_O. Each mouse was injected with 0.1 mL of THP solution each time.

The clinical tumor tissues, animal experiments, and supporting fund projects have been discussed and approved by the Ethics Committee of the Affiliated Haikou Hospital of Xiangya Medical College, Central South University. All enrolled patients and their immediate family members fully understood and agreed that the surgically removed tissues would be used for this medical research and signed the informed consent. Written informed consent has been obtained from the patients to publish this paper.

### 2.3. CRISPR/dCas9-SAM Screening Process

The T24 cells were transduced with lentivirus carrying the CRISPR-Pool™ SAM library, with a multiplicity of infection of 1. Hygromycin B selection was carried out for 2 days. Then, the transduced T24 cells were treated with THP for 14 days, and the surviving cells at the end of days 7 and 14 were harvested. Genomic DNA was extracted and amplified. Then, the sgRNA sequence in the genome of the living cells was deeply sequenced. For data analysis, the enrichment score was calculated as Formula (1) [20]. The sgRNAs used for validation were synthesized and constructed as described [21]. The screening process is shown in Figure 1B.

(1) $$\mathrm{The}\;\mathrm{enrichment}\;\mathrm{score}=\frac{0.25\times \mathrm{sgRNA}\;\mathrm{from}\;\mathrm{the}\;\mathrm{reads}}{\mathrm{sgRNA}\;\mathrm{in}\;\mathrm{the}\;\mathrm{library}}\times \log_{2}^{\mathrm{average}\;\mathrm{abundance}}$$

### 2.4. Off-Target Effect Examination

Off-target sites were predicted using an online search tool (http://crispr.mit.edu, 20 May 2021). In addition, 3 bp mismatches compared with the target consensus sequence were allowed.

### 2.5. Quantitative Real-Time Polymerase Chain Reaction (RT-PCR)

Total RNA was extracted from tissues using the RNA isolater Total RNA Extraction Reagent (Vazyme, Nanjing, China, R401-01). Reverse transcription to cDNA was performed using HiScript^®^III All-in-one RT SuperMix Perfect for qPCR (Vazyme, R333). The cDNA was amplified with an Applied Biosystems QuantStudio 5 Real-Time PCR instrument (Thermo Fisher Scientific, Waltham, MA, USA) with ChamQ Universal SYBR qPCR Master Mix (Vazyme, Q711). The primer sequences used were as follows:

- AKR1C1 forward prime: 5′-CTTCAAAGCTTTGGTGCAATTC-3′
- AKR1C1 reverse prime: 5′-ATCTTTTGGGATCACTTCCTCA-3′
- ACTB forward prime: 5′-CATGTACGTTGCTATCCAGGC-3′
- ACTB reverse prime: 5′-CTCCTTAATGTCACGCACGAT-3′

The 2^−∆∆Ct^ comparative method was applied to calculate the relative AKR1C1 mRNA expression.

### 2.6. Immunoblot Analysis

The TPEB mixed reagents were made by phosphatase inhibitor cocktail (DI201, TransGen Biotech, Beijing, China), protease inhibitor cocktail (DI101, TransGen Biotech, Beijing, China), and mammalian total protein extraction kit (TPEB) (DE101, TransGen Biotech, Beijing, China) according to the protocols. Then, the cells were mixed with the TPEB mixed reagents on ice. The lysates were collected and centrifuged at 14,000× *g* for 10 min at 4 °C. Protein concentrations were analyzed using a bicinchoninic acid (BCA) kit (P0010, Beyotime Biotechnology, Shanghai, China). Equal amounts of protein samples were separated through electrophoresis on a 10% precast gel (M00664, GenScript Biotech, Nanjing, China). The proteins were then transferred to PVDF membranes. The membranes were blocked with 5% skimmed milk overnight at 4 °C. Afterward, the membranes were incubated for 2 h at room temperature with different primary antibodies, including AKR1C1 (ab192785, 1/10,000), β-actin (ab8227, 1/10,000), Bax (ab32503, 1/10,000), Bcl-2 (ab32124, 1/10,000), caspase-9 (ab32539, 1/1000), caspase-3 (ab32351, 1/1000), caspase-8 (ab32397, 1/1000), cleaved Caspase-3 (ab32042, 1/1000), Nrf2 (ab62352, 1/5000), p-Nrf2 (ab76026, 1/1000), and KEAP1 (ab227828, 1/10,000). The second antibody was HRP goat anti-rabbit (ab6721, 1/2000–1/20,000). The above antibodies were purchased from Abcam (Cambridge, MA, USA). The membranes were washed three times with TBST and incubated with the secondary antibodies (1/2000–1/20,000) for 1 h at room temperature. The target protein lane was imaged using an iBright 1500 (Invitrogen, Carlsbad, CA, USA) enhanced by chemiluminescent substrates (Merck Millipore, Darmstadt, Germany, WBKLS0500).

### 2.7. IC50 Assay

The target cells to be tested were digested, resuspended, and evenly planted in 96-well plates. PBS was added in the outer side of the circle to prevent edge effects. Cell adherent growth after 24 h. The THP (MCE, Belleville, NJ, USA, HY-13725) solution was diluted by dissolution in DMSO into gradient concentration with FBS-free medium. At this time, the DMSO content in the highest concentration group was 0.159%. The medium in the 96-well plate was discarded, and the drug-containing medium of each concentration group was added into the 96-well plate for incubation at 37 °C and 5% CO_2_. After 24 h, the drug-containing medium was discarded, and FBS-free medium containing 10% CCK-8 (Dojindo Laboratories, CK04) was added for incubation. After 2 h, the OD450 nm absorption was detected using the enzyme marker (Bio-Rad, Hercules, CA, USA). The experimental data (mean ± standard deviation) were plotted as histograms. Dose–response curves were generated using GraphPad Prism 7 (GraphPad Software, San Diego, CA, USA) software, and the absolute 50% inhibitory concentration (IC50) was determined.

### 2.8. Hematoxylin/Eosin (HE) and Immunohistochemistry (IHC) Staining

HE staining: The tissue was embedded in paraffin and cut into a 5–7 μm sheet, spread in warm water, and then transferred to the glass to dry. First, the section was placed in xylene to remove the wax, then ethanol was used to remove the xylene, then the section was dyed with hematoxylin and eosin in turn, and then anhydrous alcohol was used to make the section transparent. Finally, 1–2 drops of neutral gum were dropped on the section and covered with cover glass.

IHC staining: IHC staining of the tissue microarray and the tissue samples for the in vivo experiments was performed in the following steps: dewaxing, blocking endogenous peroxidase, serum blocking, primary antibody binding, secondary antibody binding, DAB coloration, restaining the nucleus, and dehydration seal. The results were obtained using the H-score system, which is a scoring method widely used in immunohistochemical pathology research and can accurately evaluate the positive rate of each region [22,23].

### 2.9. Construction of AKR1C1 Overexpression Stable Transgenic Strain

The AKR1C1 overexpression stable transgenic strain was constructed in T24 cell line by embedding the overexpression plasmid with lentivirus. The vector plasmid was GV492 vector, and the sequence of elements was Ubi-MCS-3FLAG-CBh-gcGFP-IRES-puromycin. The primer sequence of the target gene fragment acquisition contained the exchange pair base, the restriction enzyme site, and the 5′-end of the target gene for PCR fishing of the target gene. The lentivirus vector plasmid was purchased from the Shanghai Genechem Gene Chemical Technology Co., Ltd. (Shanghai, China). The transfection step followed the protocol related to the reagent.

The gene acquisition sequences are given in Table 3.

### 2.10. SiRNA Interference of AKR1C1 Expression in RT4 Cells

The expression level of AKR1C1 gene in the RT4 cell line was downregulated by small interfering RNA (siRNA). The siRNA was purchased from Guangzhou RiBo™ Biotechnology Co., Ltd. (Guangzhou, China) The transfection reagent was Lipofectamine 3000 (ThermoFisher, Waltham, MA, USA, L3000015). The interference experiment steps followed the reagent-related protocol.

### 2.11. 4-Hydroxynonenal (4-HNE) Assay

4-HNE was detected by ELISA. After the culture medium was discarded and cells were washed with PBS, the cell samples tested were resuspended with lysate. The cells were broken by ultrasonic crusher on the ice 5 times, at 10 s each instance. Next, the sample was placed on ice for 30 min and then centrifuged at 12,000 rpm and 4 °C for 10 min. The supernatant was taken for testing. The experimental steps followed the 4-HNE ELISA reagent (Cusabio, Wuhan, China, E16214h) protocol. Finally, the OD450 nm absorption was detected by the enzyme marker, and the standard curve was drawn using Curve Expert v1.4 software to calculate the concentration of each sample to be measured.

### 2.12. ROS Assay

Intracellular ROS levels were measured using an intracellular ROS assay kit (Solarbio Science & Technology, Beijing, China, CA1420), and 0.5 × 10^4^ cells/mL of T24 cells or 2.5 × 10^5^ cells/mL of RT4 cells were inoculated in 6 wells plates, 2 mL/well. The cells were treated with 8 nM THP (DMSO was used in the control group) for 72 h, then washed with PBS 3 times to remove the original culture medium and treated with a 10 μM dichlorofluorescein diacetate probe for 20–30 min. The cells were washed three times with a serum-free medium, and their fluorescence intensity was measured using a fluorescence microplate reader at excitation wavelength 488 nm, emission wavelength 610 nm.

### 2.13. Colony Formation and Proliferation Assay

Colony formation assay: A total of 500 T24 cells or 1000 RT4 cells were resuspended in 0.2 mL of medium, evenly added to a 6-well plate containing 1.8 mL of 10% FBS medium, and cultured in a 5% CO_2_ incubator at 37 °C for 2 weeks. During this period, the frequency of fluid change was reduced, changing of the fluid was avoided, or the cell culture medium was changed only once. After 2 weeks, the 6-well plate was taken out, and the cells were observed using a microscope to grow in a single colony. The culture medium was discarded, the PBS was gently washed three times, and the cells were dyed with 0.5% crystal violet for 15 min. After the colonies turned to purple, the cloning rate was calculated using Formula (2).

(2) Cloning rate =×100

Cell proliferation assay: The cells in each group were resuspended with a culture medium containing 10% FBS, implanted into 96-well plate, and divided into groups of 0, 24, 48, and 72 h. After 6 h, cells showed adherent growth. At this time, the 96-well plate were taken out, and the culture medium of the 0 h group was discarded. The culture medium containing 10% CCK-8 was added and incubated for 2 h. Then, the OD450 nm absorption was detected using the enzyme marker. The above operations were repeated at 24, 48, and 72 h, and the growth curves were plotted according to the obtained values.

### 2.14. Wound Healing Assay

A total of 70 μL of T24 cells were inoculated at a density of 8 × 10^5^ cells/mL into each insert of a culture-insert 2 well (Ibidi, Grafelfing, Germany) at the middle of a dish. After the cells were attached, the insert was removed. Incubation was continued, and the cells were removed at 0, 24, and 48 h for observation under a microscope (IX71, Olympus, Tokyo, Japan) to determine whether the peripheral cells had migrated to the central scratch area. The cells were photographed and recorded. The percentage of wound healing was analyzed using the ImageJ software and calculated as the ratio of the initial scratch area minus the partially healed area that had healed at a certain time to the initial area, using Formula (3).

(3) $$\mathrm{Wound}\;\mathrm{area}\;\left(\%\right)=\left(\frac{\mathrm{area}\;\mathrm{at}\;a\;\mathrm{certain}\;\mathrm{time}}{\mathrm{initalral}\;\mathrm{area}}\right)\times 100$$

### 2.15. Transwell Assay

The T24 cells were collected and adjusted to 5 × 10^4^ cells/well. The cells were inoculated into the upper chamber of a Transwell plate, and the lower chamber was supplemented with 10% FBS medium. The plate was placed in the incubator for 24 h, washed twice with PBS, and fixed with methanol. Then, the cells were treated with 0.1% Giemsa staining solution, washed three times with PBS, and allowed to air dry. The number of migrated cells was recorded by photography under multiple high-magnification fields using a microscope (Etaluma, Inc., San Diego, CA, USA, LS720) and by counting the number of migrated cells.

### 2.16. Apoptosis Assay

Apoptosis was detected by TUNEL (Beyotime, Shanghai, China, C1090). After the original culture medium was discarded, the cells from each group were washed once with PBS. Then, the cells were fixed with an immune fixative (Beyotime, P0098) for 30 min, washed again after 30 min, added to an immune permeable solution (Beyotime, P0097), and incubated at room temperature for 5 min. The TUNEL solution was prepared according to the protocol and used to incubate cells at 37 ℃, away from light. After 1 h, the TUNEL solution was drawn, washed with PBS three times, and then restained with DAPI (Beyotime, C1002). TUNEL (red fluorescence) was detected at 550 nm, and DAPI (blue fluorescence) was detected at 360–440 nm.

Flow CytoMetry (FCM) is also used to detect apoptosis in RT4 cells. The RT4 cells were treated with different aspirin or normal saline (NC) for 24 h, then treated with THP or DMSO for 24 h, digested by using EDTA-free trypsin, collected, washed, and resuspended with PBS, and centrifuged to collect cell precipitates. The precipitates were resuspended again with a small amount of a binding buffer, mixed with an Annexin-V-FITC working solution, incubated for 5 min at room temperature under protection from light, and mixed with a PI reagent and PBS. The samples were analyzed using flow cytometry and the results were analyzed using Modfit software. Cells in the Q2 + Q4 region are generally considered apoptotic.

### 2.17. Statistical Analysis

SPSS 21.0 and GraphPad Prism 7.0 software were used for the statistical analyses. For the comparison between the two sets of data, the Student *t*-test was used for normal distribution data, and the Mann–Whitney U nonparametric test was used for non-normal distribution data. The comparison between multiple groups was conducted using one-way ANOVA. When the data met the normal distribution, the Bonferroni post hoc test was used for homogeneous variance data, and the Tamhane T2 post hoc test was used for uneven variance. The Kruskal–Wallis nonparametric test is used to compare data with a non-normal distribution among multiple groups. *p* < 0.05 was considered statistically significant.

## 3. Results

### 3.1. AKR1C1 Was Significantly Enriched by CRISPR/dCas9-SAM Genome-Wide Library

The steps of CRISPR/dCas9-SAM filtering are shown in Figure 1B. By comparing the difference of sgRNAs between the experimental (THP treatment) and the control (DMSO) groups, the candidate genes were screened positively or negatively. Positive screening indicated that the content of sgRNAs in the experimental group was higher than that in the day 0 group. By contrast, negative screening indicated that the content of sgRNAs was lower than that in the day 0 group. In this study, the genes enriched and increased after THP treatment were determined; positive screening was chosen. The expression value of the screened gene was usually represented by the Z-score. The Z-score was derived from the difference between a certain sgRNA sequence in each group and day 0 group, which reflects the intensity of gene screening. The larger the Z-score value, the more enriched the sgRNA. The expression amount of each gene after screening could be obtained by comparing the Z-score value between groups. A total of 1493 genes enriched by positive screening were obtained, and the top 50 genes are shown in the heat map (Figure 1D).

To more intuitively show the variation degree of each gene enrichment, the enrichment score [20] was used to show the top 30 genes, as shown in Figure 1C.

After the CRISPR/dCas9 SAM genome-wide library was combined with THP positive screening, AKR1C1 gene was found to be the most significantly enriched between the THP and control DMSO groups on day14, and the expression multiple in THP group was 10.298 times that of the control group. Therefore, among all candidate genes, AKR1C1 had the most potential to become the target gene selected in this study.

### 3.2. AKR1C1 Has Different Expression in Different Bladder Cancer Cell Lines or Clinical Tissues

**Figure 2:**
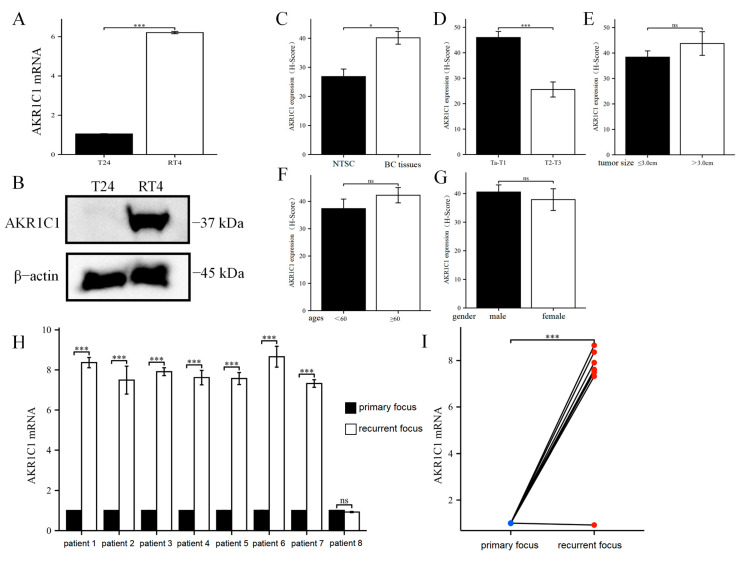
AKR1C1 has different expressions in different bladder cancer cell lines or clinical tissues. (**A**) Difference of AKR1C1 gene expression between T24 and RT4 cells. (**B**) The expression difference of AKR1C1 protein between T24 and RT4 cells. (**C**–**G**) The expression of AKR1C1 in tissue microarray was different in various patient clusters. (**H**,**I**) The expression level of AKR1C1 gene in the tumor tissue of primary and recurrent bladder cancer of eight patients in our cohort. AKR1C1: aldo-keto reductase family 1 member C1, BC: bladder cancer, NTSC: normal tissue surrounding cancer. 2^−∆∆Ct^ is used in the analysis of qRT-PCR assay. The data were expressed as mean ± S.D, *n* = 3; * *p* < 0.05, *** *p* < 0.001, ns: no statistical difference. The uncropped blots are shown in Figures S2 and S3.

First, the expression level of AKR1C1 was detected in the T24 and RT4 cell lines at the mRNA and protein levels, and the results are shown in Figure 2A,B. The expression of AKR1C1 in the T24 cell line was significantly lower than that in the RT4 cell line at both levels.

Second, the sites in the tissue microarray with AKR1C1 were stained by IHC, and the results are shown in Figure 2C–G. AKR1C1 expression in the cancer tissue was significantly higher than that in the normal tissue surrounding cancer and was significantly higher in the Ta/T1 bladder cancer tissue than that at the T2/T3 stage. However, no statistical difference was found between the AKR1C1 expression level and tumor size, age, and gender.

Finally, the expression level of AKR1C1 was measured in the bladder cancer tissues of eight clinical patients from the center at the time of initial onset and recurrence. The clinical data of these patients are shown in Table 2. All patients had received THP treatment, and bladder tumor recurrent occurred within 5–28 months after treatment. As shown in Figure 2H,I, the expression levels of AKR1C1 in the recurrent bladder cancer foci of seven of eight patients were significantly higher than that in the primary focus, and only one patient had no significant difference between the expression of AKR1C1 in the recurrent and the primary focus.

### 3.3. AKR1C1 Expression Level Is Positively Correlated with IC50 to THP in Bladder Cancer Cells

**Figure 3:**
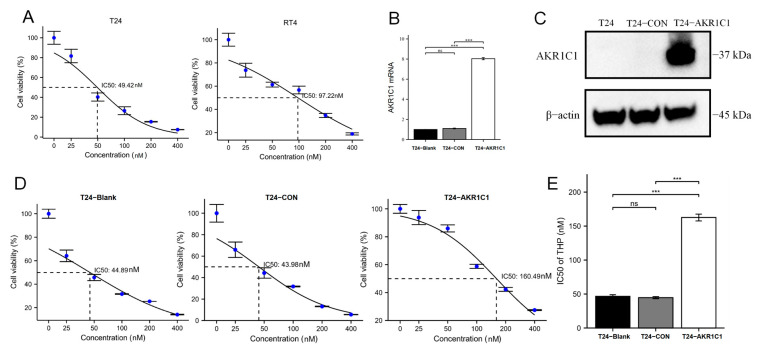
Overexpression of AKR1C1 in T24 cells could induce resistance to THP. (**A**) IC50 of T24 and RT4 cell lines to THP, respectively. (**B**,**C**) T24-AKR1C1 overexpression stable transgenic cells express AKR1C1 in both genes and proteins. (**D**,**E**) Overexpression of AKR1C1 increased the resistance of T24 cells to THP. AKR1C1: aldo-keto reductase family 1 member C1, CON: negative control group, THP: pirarubicin. 2^−∆∆Ct^ is used in the analysis of qRT-PCR assay. The data were expressed as mean ± S.D, *n* = 3; *** *p* < 0.001, ns: no statistical difference. The uncropped blots are shown in Figures S4 and S5.

The IC50 of T24 and RT4 cell lines to THP were detected to be 46.59 ± 2.01 nM and 94.36 ± 2.27 nM, respectively (Figure 3A). Afterward, the T24 cell line were transfected with lentivirus embedding plasmid to overexpress AKR1C1, and a stable transfection strain was screened out through puromycin (Supplementary Figure S1). Then, the expression level of AKR1C1 was detected at the mRNA and protein levels, as shown in Figure 3B,C. The expression level of AKR1C1 in T24-AKR1C1 overexpression stable transgenic strain was significantly higher than those in the blank group (T24) and negative control group (T24-CON) at both levels. The IC50 (162.62 ± 4.14 nM) of T24-AKR1C1 stable transfection to THP was also significantly higher than those of the blank group (46.59 ± 2.01 nM) and T24-CON (44.72 ± 1.29 nM) (Figure 3D,E).

**Figure 4:**
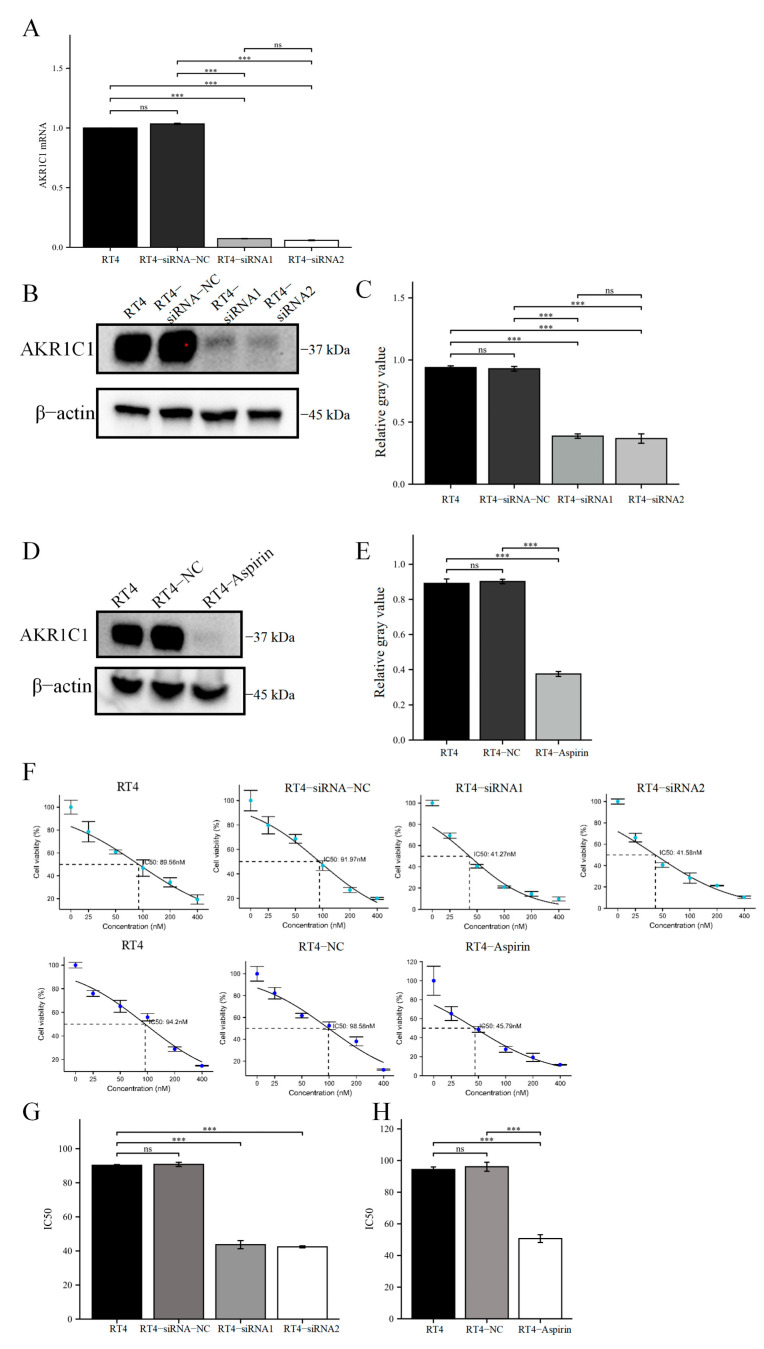
Interference or inhibition of AKR1C1 could reduce the resistance of RT4 cells to THP. (**A**–**C**) The expression of AKR1C1 in RT4 cells was significantly inhibited by gene and protein after siRNA interference. (**D**,**E**) Aspirin significantly inhibits AKR1C1 protein in RT4 cells. (**F**–**H**) The IC50 of RT4 cells with siRNA interference and aspirin treatment to THP, respectively. AKR1C1: aldo-keto reductase family 1 member C1, CON: negative control group, THP: pirarubicin. 2^−∆∆Ct^ is used in the analysis of qRT-PCR assay. The data were expressed as mean ± S.D, *n* = 3; *** *p* < 0.001, ns: no statistical difference. The uncropped blots are shown in Figures S6–S9.

In the RT4 cell lines, siRNA and aspirin were used to reduce the expression level of AKR1C1. After siRNA interference, AKR1C1 expressed by RT4 was significantly inhibited at the mRNA (Figure 4A) and protein levels (Figure 4B,C). In addition, acetylsalicylic acid, also known as aspirin, is a common non-steroidal anti-inflammatory drug, but it is also an inhibitor of AKR1C1, which can effectively inhibit the activity of AKR1C1 [24,25]. After using aspirin, the AKR1C1 expression in RT4 at the protein level was also significantly inhibited (Figure 4D,E). Afterward, the IC50 of THP was detected between cells in each group. The results showed that siRNA interference with the expression of AKR1C1 could reduce the IC50 of RT4 cells to THP (Figure 4F,G). After siRNA-1 interference, the IC50 of the RT4 cell lines to THP was 43.68 ± 3.37 nM, while the siRNA-2 group was 42.39± 0.84 nM. The blank and negative control groups were 90.27 ± 0.61 nM and 90.78 ± 1.72 nM, respectively. Using aspirin also reduced the IC50 of RT4 cell line to THP, as shown in Figure 4F,H. The IC50 of RT4 cell line to THP in the aspirin group was 50.67 ± 3.48 nM, significantly lower than those of the blank (RT4) (94.36 ± 2.27 nM) and negative control groups (RT4-NC) (96.08 ± 4.03 nM).

### 3.4. The Expression Level of AKR1C1 Did Not Affect the Proliferation, Invasion, or Migration of the Bladder Cancer Cells

**Figure 5:**
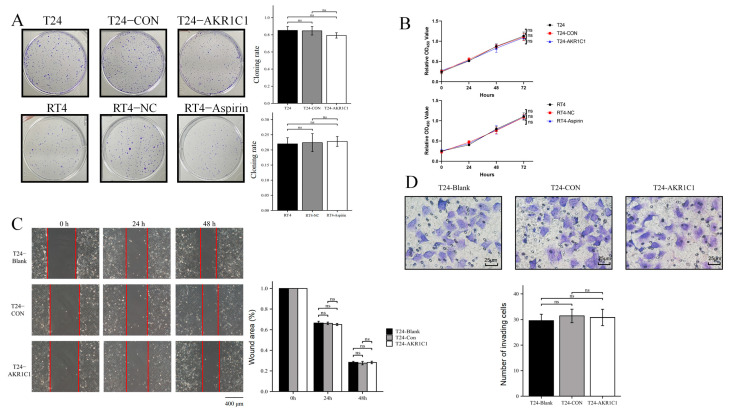
The expression level of AKR1C1 did not affect the proliferation, invasion, or migration of the bladder cancer cells. (**A**,**B**) Overexpression or inhibition of AKR1C1 does not affect cell colony formation (**A**) and proliferation (**B**) assay in bladder cancer cells. (**C**,**D**) Overexpression of AKR1C1 did not affect the wound healing (**C**) and the Transwell assay (**D**) of T24 cells. The data were expressed as mean ± S.D, *n* = 3, while *n* = 5 for the Transwell assay; ns: no statistical difference.

The effects of AKR1C1 on the proliferation and colony formation on the bladder cancer cells were tested. Overexpression of AKR1C1 had no significant effect on cell colony formation ability in the T24 cells (Figure 5A). The use of aspirin to inhibit the function of AKR1C1 in RT4 cells also had no significant effect on the ability of colony formation. In the cell proliferation assay, regardless of whether AKR1C1 was overexpressed in T24 or AKR1C1 was inhibited in RT4, no significant effect was observed on the proliferation in the bladder cancer cells (Figure 5B).

In addition, wound healing and the Transwell assay showed that overexpression of AKR1C1 in T24 cells had no effect on the migration and invasion in the bladder cells (Figure 5C,D).

In this part, only T24 cell line was used to carry out wound healing and the Transwell assay, because RT4 cells had poor migration and invasion ability. Thus, the two experiments were generally not carried out in RT4. In addition, the RT4 cell line was not used with siRNA interference in the cell cloning and proliferation assay, because the phenotype after siRNA interference could only be maintained for 72–96 h. Beyond this time range, the experimental results may be unreliable, while the cloning and proliferation assay experiment takes a long time.

### 3.5. Overexpression of AKR1C1 Can Reduce THP Induced 4-HNE and ROS Production and Inhibit Apoptosis of Bladder Cancer Cells

As an aldehyde and ketone reductase, AKR1C1 can reduce the production of 4-HNE and inhibit the production of ROS by degrading quinones in anthracycline drugs to hydroquinones. 4-HNE is the main active aldehyde produced by lipid peroxidation and an important intermediate product under oxidative stress. 4-HNE accumulates in cells and destroys the balance of mitochondria, thus promoting ROS production and causing cell apoptosis [26].

**Figure 6:**
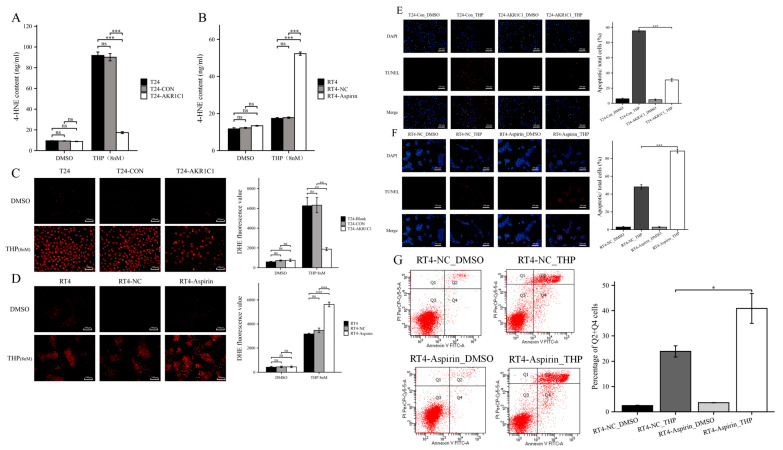
Overexpression of AKR1C1 reduces THP-induced 4-HNE and ROS, and resists apoptosis in T24 cells. (**A**,**C**) Overexpression of AKR1C1 reduced THP-induced 4-HNE and ROS in T24 cells. (**B**,**D**) Inhibition of AKR1C1 increases THP-induced 4-HNE and ROS in RT4 cells. (**E**) Overexpression of AKR1C1 resists THP-induced apoptosis in T24 cells. (**F**) Inhibition of AKR1C1 enhances THP-induced apoptosis in RT4 cells. (**G**) Flow cytometry detected that the number of apoptotic cells in RT4 cells treated with aspirin increased after THP treatment. 4-HNE: 4-hydroxynonenal, AKR1C1: aldo-keto reductase family 1 member C1, DMSO: dimethyl sulfoxide, THP: pirarubicin. Three independent experiments were completed for each assay. The data were expressed as mean ± S.D, *n* = 3; * *p* < 0.05, ** *p* < 0.01, *** *p* < 0.001, ns: no statistical difference. Each scale bar = 200 μm.

After 72 h of treatment with THP (8 nM), the content of 4-HNE (17.32 ± 1.36 ng/mL) in the T24-AKR1C1 overexpression stably transfected cells was significantly lower than those in the blank group (91.87 ± 4.68 ng/mL) and the negative control group (90.14 ± 4.96 ng/mL) (Figure 6A). Similarly, after 72 h of THP treatment, the intracellular 4-HNE content (52.28 ± 1.27 ng/mL) in the RT4-aspirin group was significantly higher than those in the blank group (17.38 ± 0.61 ng/mL) and negative control group (17.76 ± 0.53 ng/mL) (Figure 6B). This result indicated that after THP treatment, the high expression of AKR1C1 led to the decrease of 4-HNE content in the human bladder cancer cells.

Then, the levels of ROS in the cells of each group after THP treatment were detected. The results are shown in Figure 6C,D. After 72 h of THP treatment, the ROS level in the T24-AKR1C1 overexpression stably transfected cells was significantly lower than those in the blank group and negative control group. The ROS level in the RT4 cells treated with aspirin was significantly higher than those in the blank and the negative control groups.

The apoptosis induced by THP was detected in each group of cells. TUNEL assay was used to mark all apoptotic cells with red fluorescence, while DAPI assay was used to mark the nuclei of all cells with blue fluorescence. The ratio of apoptotic cells was obtained by calculating the ratio of TUNEL positive cells to the total number of cells. As shown in Figure 6E,F, THP could significantly induce apoptosis in the blank and negative control groups, but the ability of THP to induce apoptosis was significantly blocked in the experimental group (T24-AKR1C1). The ratio of apoptotic cells was 95.573% ± 2.292% in the negative control group, while the ratio of apoptotic cells was 30.805% ± 2.567% in the experimental group. Similarly, RT4 cells became more sensitive to THP-induced apoptosis after being treated with aspirin. The ratio of apoptotic cells was 48.079% ± 3.667% in the negative control, while the ratio of apoptotic cells was 88.372% ± 3.166% in the aspirin-treated group. In addition, flow cytometry testing found that using aspirin to inhibit AKR1C1 significantly increased the level of THP induced apoptosis (Figure 6G).

**Figure 7:**
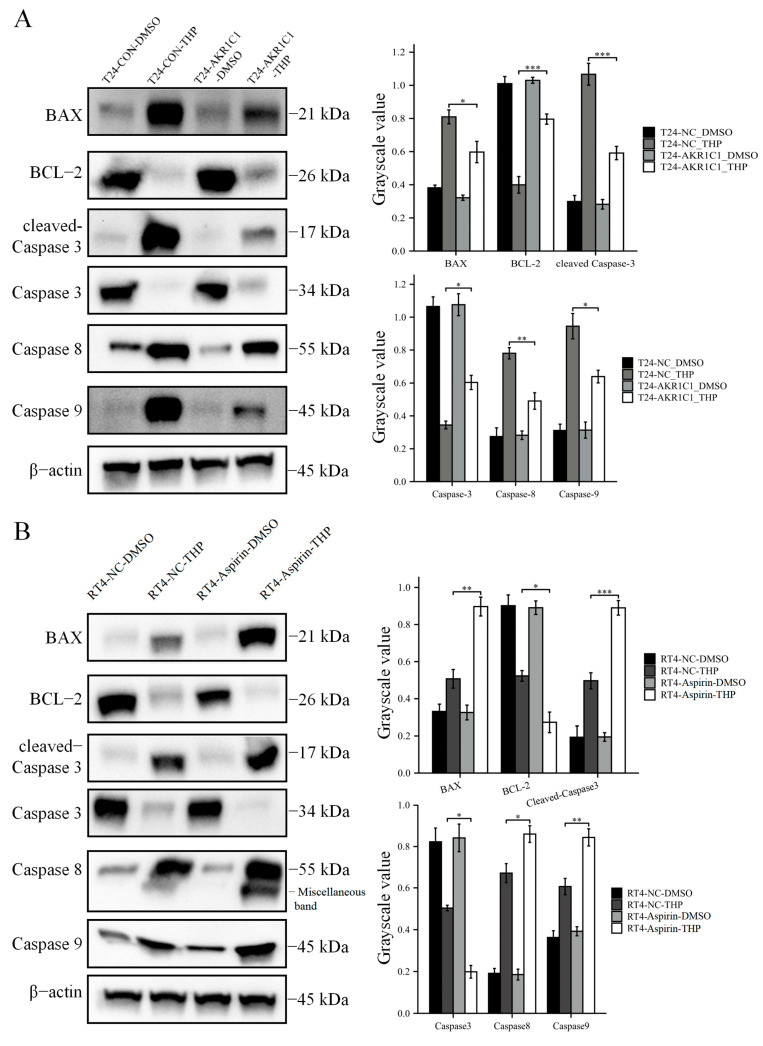
The expression level of AKR1C1 affects the expression of apoptosis-related proteins induced by THP in bladder cancer cells. (**A**) Overexpression of AKR1C1 inhibits THP-induced apoptosis-related proteins in T24 cells. (**B**) Inhibition of AKR1C1 increases THP-induced apoptosis-related proteins in T24 cells. AKR1C1: aldo-keto reductase family 1 member C1, BAX: BCL-2 associated X, BCL-2: B-cell lymphoma-2, Caspase: cysteine aspartate-specific protease, DMSO: dimethyl sulfoxide, THP: pirarubicin. The data were expressed as mean ± S.D, *n* = 3; * *p* < 0.05, ** *p* < 0.01, *** *p* < 0.001, ns: no statistical difference. The uncropped blots are shown in Figures S10–S23.

Finally, the expression of apoptosis-related proteins among cells in each group was detected by immunoblot, and the results are shown in Figure 7. In the T24 bladder cancer cell line (Figure 7A), THP treatment significantly increased the expression of apoptosis-related proteins, such as BAX, cleaved caspase-3, caspase-8, and caspase-9, while the expression level of anti-apoptotic protein BCL-2 was inhibited. THP promoted the apoptosis of T24 cells of bladder cancer. However, after overexpression of AKR1C1 gene in the T24 cells and THP treatment, the expression level of apoptosis-related protein decreased compared with the control group, while the expression level of anti-apoptotic protein BCL-2 increased significantly. Total caspase-3 was activated under the induction of THP and cut itself to produce cleaved-caspase3, resulting in a decline in its own level. However, the expression of caspase-3 in the T24-AKR1C1 group was slightly higher than that of the negative control group. The overexpression of AKR1C1 gene in the T24 cells has been suggested to reduce the activation of caspase-3 and resist THP-induced apoptosis. The expression of apoptosis-related proteins in RT4 was detected similarly (Figure 7B). In the RT4 cells, THP treatment significantly promoted the expression of apoptosis-related proteins in the RT4 cells, such as BAX, cleaved caspase-3, caspase-8, and caspase-9. These proteins were further upregulated after treatment with aspirin. The expression of antiapoptotic protein BCL-2 was significantly inhibited in the aspirin group compared with the control group, and the expression level of total caspase-3 was reduced. This result indicated that aspirin could synergistically promote the apoptosis of THP in the RT4 cells.

### 3.6. THP Could Upregulate the Expression of AKR1C1 in the T24 Cells via the ROS/KEAP1/NRF2 Pathway and Tempol Could Block It

Nuclear factor E2 related factor-2 (NRF2) is a highly conserved transcription factor with alkaline leucine zipper, responsible for regulating the transcription of multiple genes. AKR1C1 is one of the target genes to be regulated. In physiological conditions, NRF2 will be stably bound by Kelch-like epichlorohydrin associated protein-1 (KEAP1) and occupy its active subunit, so it does not exhibit transcriptional activity. During severe oxidative stress and a large amount of ROS accumulation in cells, KEAP1 uncouples with NRF2, thus releasing NRF2 with transcriptional activity, and then regulating the transcription of many antioxidant stress genes including AKR1C1. After the oxidative stress disappears, NRF2 is re-bound by KEAP1 again, inhibiting its transcription activity and ending the antioxidant stress response [27,28]. In addition, free NRF2 can also be phosphorylated, and phosphorylated NRF2 will lose its binding site with KEAP1, which can stably and continuously promote gene transcription related to antioxidant stress in the nucleus [27].

**Figure 8:**
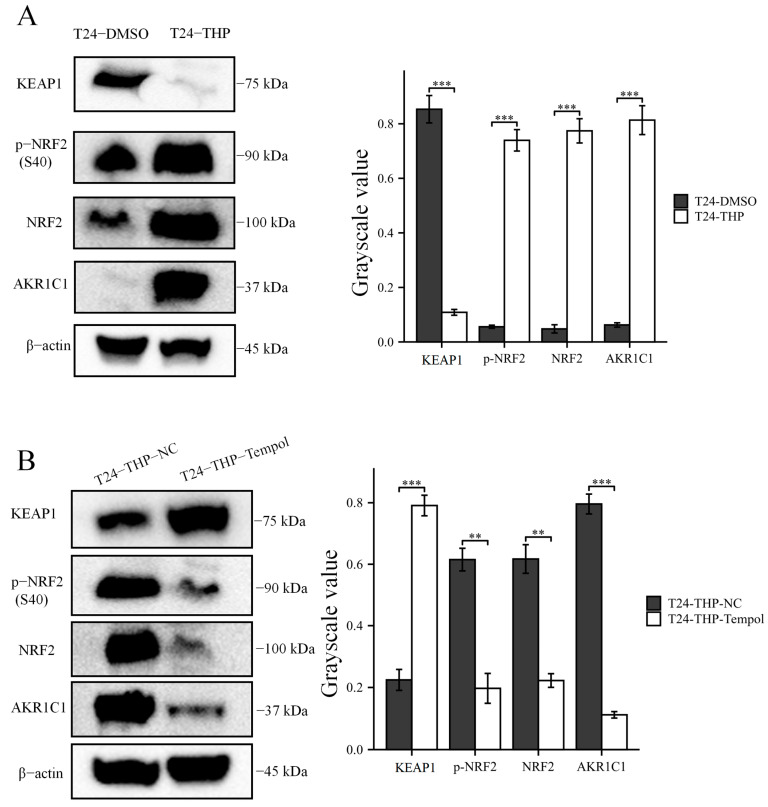
THP could upregulate the expression of AKR1C1 in the T24 cells via the ROS/KEAP1/NRF2 pathway and tempol could block it. (**A**) After THP treatment, AKR1C1 expression increased via KEAP1/NRF2 pathway. (**B**) Tempol can block the increase of AKR1C1 expression after THP treatment. AKR1C1: aldo-keto reductase family 1 member C1, KEAP1: kelch-like epichlorohydrin associated protein-1, NRF2: nuclear factor E2 related factor-2, p-: phosphorylated. The data were expressed as mean ± S.D, *n* = 3; ** *p* < 0.01, *** *p* < 0.001. The uncropped blots are shown in Figures S24–S33.

A small dose of THP (4 nM) was used to maintain the culture of T24 cells for 1 week (0.14% DMSO was used in the control group), and the fluid was changed every 3 days. Simulating NMIBC patients receiving THP perfusion therapy may reveal the mechanism that non-drug-resistant patients with low initial AKR1C1 expression have increased expression and promoted drug resistance after a certain number of treatments. The expression of KEAP1 in the T24 cells decreased, while the expression of p-NRF2 (S40) and NRF2 increased, and the expression of AKR1C1 was significantly upregulated (Figure 8A).

Then, 3 mM tempol was used to scavenge ROS induced by THP. Tempol is a water-soluble superoxide dismutase (SOD) analog that can effectively neutralize ROS produced in the cells [29]. As shown in Figure 8B, the expression of p-NRF2, NRF2, and AKR1C1 in the tempol group was significantly inhibited, while the expression of KEAP1 was upregulated. Thus, tempol could save the upregulation of AKR1C1 gene expression induced by THP to a certain extent. Therefore, THP regulated AKR1C1 expression through KEAP1/NRF2 by stimulating ROS production.

### 3.7. AKR1C1 Could Also Cause Resistance of Bladder Cancer Cells to THP In Vivo

**Figure 9:**
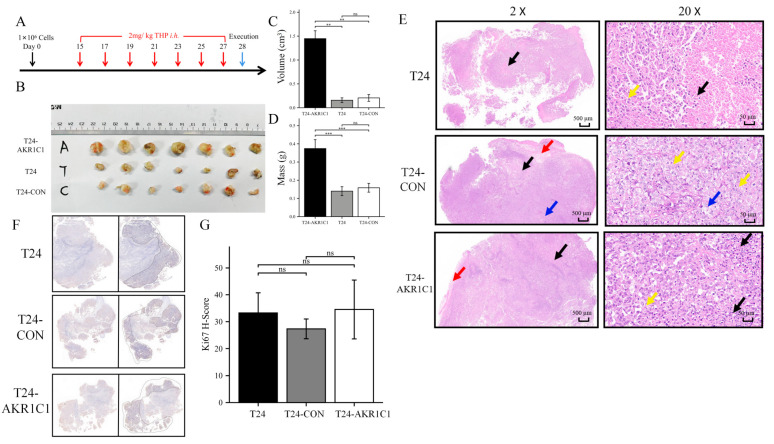
AKR1C1 could also cause resistance of bladder cancer cells to THP in vivo. (**A**) Scheme of in vivo experiment. (**B**) Gross specimen of subcutaneous transplanted tumor. (**C**,**D**) The difference between the volume (**C**) and mass (**D**) in the three groups of tumors. (**E**) HE staining of three groups of tumor sections. (**F**,**G**) Ki67 IHC staining and statistical results of three groups of tumor sections. AKR1C1: aldo-keto reductase family 1 member C1, HE: hematoxylin-eosin. Yellow arrow: mitotic phenomenon, black arrow: enhanced eosinophilic cytoplasm, blue arrow: infiltration of lymphocytes or granulocytes in the interstitial, red arrow: infiltration of lymphocytes or granulocytes in surrounding connective tissue. ** *p* < 0.01, *** *p* < 0.001, ns: no statistical difference.

A total of 30 BALB/c nude mice (aged 4–6 weeks and in good condition) were selected and randomly divided them into three groups, with five males and five females in each group. The first group was inoculated with T24 bladder cancer cell line (blank control group). The second group was inoculated with T24-CON negative control stable transfer strain (negative control group). The third group was inoculated with T24-AKR1C1 overexpression stable transgenic strain. Each mouse was injected with 0.1 mL (1 × 10^7^ cells/mL) cell suspension at left armpit. The tumor formation was observed by grouping and cage feeding. After 2 weeks, the tumorigenic mice in all groups were treated with THP. The treatment scheme was administration of THP by hypodermic injection at 2 mg/kg. The injection was administered by local cross injection around the tumor once every other day, for 14 days. After the treatment, the nude mice were killed by cervical dislocation, the tumor tissue was taken out. The tumor size was measured and weighed according to the grouping. The experimental process is shown in Figure 9A.

In the three groups of xenotransplantation nude mice tumorigenesis models, seven mice in each group had tumorigenesis and survived until the end of the experiment. In the T24 group, four males and three females mice survived. In the T24-CON group, two males and five females survived. In the T24-AKR1C1 group, three males and four females survived.

After measuring and weighing the tumors in the nude mice, the responsiveness of the three groups of mouse tumors to THP were tentatively obtained. The results are shown in Table 4 and Table 5 and Figure 9B–D. The tumor tissue volume and mass of the experimental group (overexpression of AKR1C1 genome) were significantly higher than those of the blank and the control group after receiving THP treatment. In the experimental group, the mass of the tumor tissue after THP treatment was 0.374 ± 0.120 g, and the volume was 1.448 ± 0.403 cm^3^. The masses of the blank and control groups were 0.14 ± 0.06 g and 0.173 ± 0.082 g, respectively, while the corresponding volumes were 0.158 ± 0.121 cm^3^ and 0.206 ± 0.173 cm^3^.

Then, HE and IHC staining were performed after three tissue wax sections were randomly taken from each group. The HE staining results are shown in Figure 9E. The three groups of sections showed that the tumor tissue structures were loose, and the cell arrangement was irregular. The tumor cells were irregular in shape, with a high ratio of nucleus to cytoplasm, heterotypic nucleus, and occasional mitotic phenomenon (yellow arrow), accompanied by varying degrees of necrosis, nuclear fragmentation, enhanced eosinophilic cytoplasm (black arrow), and infiltration of lymphocytes or granulocytes in the interstitial (blue arrow) and surrounding connective tissue (red arrow). However, in the blank and control groups, large areas of necrosis, accompanied by a large number of tumor cell necrosis and nuclear fragmentation, were observed. By contrast, in the experimental group with overexpression of AKR1C1, only multifocal necrosis, a small number of tumor cell necrosis, and nuclear fragmentation were noted.

The IHC staining results are shown in Figure 9F,G. The IHC staining of Ki67 were completed in three groups of sections, and the H-score system was adopted to evaluate the positive rate. No significant difference was found between the three groups in the H-score of Ki67, indicating no significant differences in the expression levels of Ki67 in the three groups of tumor tissue.

## 4. Discussion

Chemotherapy resistance of bladder cancer is one of the most serious problems in clinical practice. The recurrence or progression of bladder cancer caused by drug resistance accounts for more than half of the total recurrence and progression. This phenomenon has become one of the important factors that restrict the prognosis and quality of life of patients with bladder cancer. THP is one of the most commonly used intravesical chemotherapy drugs for patients with NMIBC. However, THP resistance or tumor recurrence after the clinical THP treatment has been increasingly reported. CRISPR/dCas9 SAM library screening technology is an efficient, rapid, and accurate method for screening genes. At present, this technology has been widely used to screen the drug- or toxicant-resistant genes of pathogenic microorganisms and plants. However, this research method has not been widely applied in basic medicine, especially in tumor drug resistance. Its application on bladder cancer-related fields has not been reported yet. The full-text database screening method based on the CRISPR/Cas9 system has caused a technological revolution in biomedical research since it was discovered and used in basic research [30]. This strategy has helped researchers worldwide to systematically verify the key genes and mechanisms behind a series of biochemical and molecular biological processes, including finding drug-sensitive genes [31,32], virus infection mechanism [33,34], and tumor proliferation and metastasis [35]. Goodspeed et al. [36] reported that MSH2 gene played a crucial role in cisplatin-mediated cell death in myometrial invasive bladder cancer. A CRISPR library screening strategy was also used in this study. These successful applications showed that CRISPR library screening will greatly promote the understanding of basic biological processes. However, given that the core principle of CRISPR/Cas9 was to knock out the target sequence, the MSH2 gene screened by Andrew et al. was actually a gene that mediated bladder cancer to be more sensitive to cisplatin, rather than a drug-resistant gene. Nevertheless, this study was the first to use the whole genome CRISPR/dCas9 SAM library in bladder cancer to find the mechanism behind pirarubicin resistance. Compared with previous studies, completely different findings were obtained by using the SAM library combined with THP to further screen the gene expression profile data of T24 bladder cancer cells. Through the screening of the CRISPR/dCas9 SAM library, 1493 genes that were positively screened were identified after THP treatment. Analysis of the difference in gene expression showed that AKR1C1 was the most obvious gene enriched on the 14th day of THP treatment. AKR1C1 gene is an aldehyde and ketone reductase that is widely distributed from lower prokaryotes to the human body and deeply involved in the metabolic regulation of various substances. In addition, AKR1C1 has been reported in various tumors that may lead to the tolerance of tumor cells to chemotherapy drugs, such as anthracycline compounds. For example, Han et al. [37] identified that AKR1C1 was one of the genes that led to the resistance of glioblastoma to adriamycin through transcriptome sequencing. In addition, the results showed that AKR1B1, AKR1C2, and AKR1C3, which were also members of the aldosterone reductase family, were also involved in the mechanism of the resistance of glioblastoma to adriamycin. The overexpression of AKR1C1 was detected in both doxorubicin-resistant lung cancer cells [38] and epirubicin-resistant breast cancer [39]. These reports indicated that the proposed screening strategy for key genes of pirarubicin resistance in bladder cancer may be correct and effective. Through the study of tissue microarray and IHC methods, AKR1C1 was found to be significantly higher in the bladder cancer tissues than in adjacent tissues, which indicated that AKR1C1 was originally high in the bladder cancer tissues at the protein level. Therefore, compared with normal adjacent tissues, the high level of AKR1C1 in bladder cancer tissues was more capable of eliminating the anticancer activity of multiple drugs and gained certain advantages for the chemotherapy resistance of bladder cancer. In addition, the AKR1C1 content in the Ta/T1 bladder cancer tissue was higher than those in the T2/T3 patients. According to current clinical guidelines, only patients in Ta and T1 may receive pirarubicin single-drug infusion therapy, while patients in T2/T3 need to face a combination chemotherapy regimen based on cisplatin. Thus, patients in Ta/T1 may face more severe challenges in pirarubicin resistance. Among the eight patients with recurrent bladder cancer, in one patient, the expression of AKR1C1 in the tumor tissue at the time of recurrence was not significantly different from that at the time of initial onset. By contrast, in the remaining seven patients, the expression of AKR1C1 in the recurrent tumor tissue was significantly higher than those in the initial tumor tissue.

Then, the original expression level of AKR1C1 was changed in the different bladder cancer cell lines by overexpression of the lentivirus plasmid, interfering with siRNA or using inhibitors. Overexpression of AKR1C1 could increase the resistance of T24 bladder cancer cell lines to THP, while interference or inhibition of AKR1C1 could increase the sensitivity of RT4 bladder cancer cell lines to THP. However, the expression level of AKR1C1 would not affect the proliferation, invasion, or migration of bladder cancer cells. Overexpression of AKR1C1 could promote bladder cancer cell lines to resist THP-induced apoptosis, while inhibition of AKR1C1 could make bladder cancer cells more susceptible to THP treatment and apoptosis. This phenomenon may be related to the AKR1C1 reduction in 4-HNE and ROS levels. The long-term treatment of THP could upregulate the expression of AKR1C1 in T24 cells, which was related to the uncoupling of KEAP1/Nrf2 and the phosphorylation of Nrf2 caused by the increase of ROS after T24 treatment. The use of the ROS scavenger tempol, a SOD analog, can significantly save the THP-induced upregulation of AKR1C1 expression.

The BALB/c nude mice were randomly divided into three groups and subcutaneously transplanted with T24 bladder cancer cell line, T24-CON negative control cell line, and T24-AKR1C1 overexpression stable strain. Tumors were successfully formed and treated with THP. After 14 days of treatment, by comparing the mass and volume of tumor tissue in the three groups, the mass and volume of the tumor tissue in the AKR1C1 stable transfection group were significantly higher than those in the blank and the negative control groups. The AKR1C1 gene was shown to promote the drug resistance of tumor tissue to THP in vivo, which was similar to the results in vitro. After HE staining of the sections of the three groups of tumor tissues, the area of cell death in the AKR1C1 overexpression group was lesser than those in the blank and negative control groups after THP treatment. This difference reflected that the experimental group had better resistance to THP compared with the tumor tissue of the blank and the control groups and was more resistant to the cell death induced by THP. These results further showed that the overexpression of AKR1C1 could enhance the drug resistance of tumor tissue to THP in xenotransplantation mice in vivo. Ki67 is an important molecular marker for detecting the proliferative capacity of solid tumors [40,41]. The expression of Ki67 in NMIBC was significantly related to the grade, stage of tumor tissue, and the size and number of tumors [42]. The results from the sections of tumor tissue in vitro and Ki67-IHC staining showed no statistical difference in the expression of Ki67 in the transplanted tumors of three groups of nude mice. The expression of AKR1C1 in the animal tissues did not cause a change in the Ki67 expression. Therefore, AKR1C1 would not promote the proliferation of tumor in vivo. These experimental results were similar to the conclusions in vivo.

**Figure 10:**
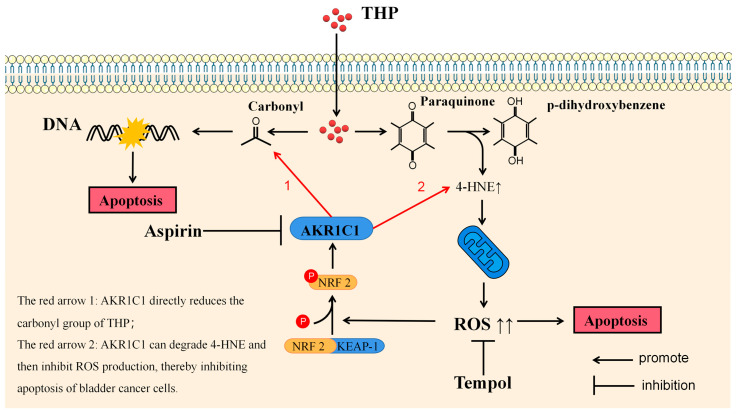
The red arrow in the figure represents two mechanisms of AKR1C1: 1. AKR1C1 directly reduces the carbonyl group of THP, thereby destroying the anticancer activity of the drug; 2. AKR1C1 can degrade 4-HNE and then inhibit ROS production, thereby inhibiting apoptosis of bladder cancer cells. 4-HNE: 4-hydroxynonenal, AKR1C1: aldo-keto reductase family 1 member C1, DNA: deoxyribonucleotide, KEAP1: kelch-like epichlorohydrin related protein-1, NRF2: nuclear factor erythroid 2 related factor 2, P: phosphorylation, ROS: reactive oxygen species.

THP, as the representative drug of anthracyclines, has been proved to participate in the antitumor effect through various mechanisms. The most well-known anticancer pharmacological action of anthracycline drugs is to exert an anticancer effect by inhibiting the interference of DNA polymerase with the synthesis of DNA in cells, causing cell cycle arrest, and promoting tumor cell apoptosis. This effect depends on the carbonyl group in pirarubicin. In the final analysis, the anticancer effect of anthracycline drugs or the side effect represented by cardiotoxicity are all due to the carbonyl group in the structure [43]. The metabolic inactivation of anthracycline drugs usually refers to their carbonyl reduction pathway. Another anticancer activity of anthracycline drugs is derived from the paraquinone (p-quinone) in the structure. P-quinone can be reduced to hydroquinone under the action of some reductases in the cell, and the surplus electrons are combined and transferred by various electrophilic groups in the cell. Finally, 4-HNE can be produced [44]. 4-HNE is a type of aldehyde substance that can destroy the potential balance of mitochondrial inner and outer membrane and promote the increase of ROS after accumulation in tissues and cells, leading to tissue cell damage. ELISA results showed that THP could significantly increase 4-HNE and ROS after acting on T24 or RT4 cells of bladder cancer. However, if AKR1C1 is overexpressed in T24 cells, the elevated levels of 4-HNE and ROS could be significantly reduced. Thus, if the inhibitor was used in RT4 to inhibit the expression of AKR1C1, 4-HNE and ROS levels were promoted. This phenomenon was also related to the enzyme function of AKR1C1 itself, because AKR1C1 was an aldehyde and ketone reductase that could reduce aldehydes or ketones (both of which have a carbonyl). AKR1C1 could open the carbon–oxygen double bond of carbonyl, thus reducing a variety of endogenous and exogenous substances, such as 4-HNE, and protecting cells from the stress caused by these substances [39]. Thus, AKR1C1 could destroy the structure of THP and its metabolites in two ways. The first one is the direct reduction of the carbonyl in the structure of THP, thereby destroying the function of THP insertion in DNA to inhibit DNA replication and promote cancer cell apoptosis. The second one is to reduce 4-HNE and inhibit its function of inducing ROS production to avoid cell apoptosis caused by ROS (Figure 10). In the current study, the expression of apoptosis-related proteins among apoptosis-treated cells were detected by TUNEL and immunoblot assay. The results confirmed that high levels of AKR1C1 could inhibit the ability of THP to induce apoptosis in bladder cancer cells, thus causing bladder cancer cells to be resistant to THP. AKR1C1 inhibitor could enhance the ability of THP to induce apoptosis in bladder cancer cells, improve the anticancer ability of THP, promote tumor cell apoptosis, and reduce drug resistance of tumor cells.

This study also has some extended problems or limitations that require further study. This study was restricted by the complexity of the pathological type, grading, staging, and treatment methods of bladder cancer and the constraints of the research methods, tools, and sample size. The authors expect to increase the sample size and conduct prospective tracking analysis of the impact of AKR1C1 gene expression level on pirarubicin treatment in the future. In addition, whether AKR1C1 can be used to predict the prognosis of NMIBC or as a target for targeted treatment will be evaluated to explore the targeted treatment drugs and means for NMIBC. In this study, aspirin and tempol were used. Aspirin is a drug widely used clinically, and tempol has been used in many clinical trials to observe its antitumor effect. More in-depth research on these two drugs will be conducted in the future to find ways to prevent or reverse the drug resistance of NMIBC to pirarubicin. Due to the anatomical characteristics of the bladder and the tissue specificity of bladder cancer, the authors will build an animal model of bladder carcinoma in situ for a more accurate, objective, and comprehensive evaluation of the impact of AKR1C1 on the proliferation, invasion, and drug resistance of bladder cancer.

## 5. Conclusions

The AKR1C1 gene determined through positive screening of the CRISPR/dCas9 SAM library could promote the human T24 bladder cancer cell line to develop resistance to THP in vivo and in vitro. Interference with AKR1C1 gene expression or the use of AKR1C1 inhibitor could significantly increase the sensitivity of the human RT4 bladder cancer cell line to THP and reduce drug resistance. However, the expression level of AKR1C1 would not change the proliferation, invasion, or migration of bladder cancers. Overexpression of AKR1C1 could inhibit THP-induced apoptosis of bladder cancer cells by inhibiting the production of 4-HNE and intracellular ROS. This process could be saved by interfering with or suppressing AKR1C1. THP treatment could upregulate AKR1C1 expression through the ROS/KEAP1/Nrf2 axis, which could be inhibited by tempol. In clinical tissues, the AKR1C1 content in the bladder cancer tissues was significantly higher than that that in the normal adjacent tissues, while it was higher in the Ta/T1 tissues than in the T2/T3 tissues. However, no relation was found among the patient’s gender, age, and the size of the primary tumor. Moreover, AKR1C1 mRNA levels in recurrent bladder cancer tissues treated with pirarubicin were higher than those in the NMIBC tissues initially found.

## Figures and Tables

**Table 1:** Information of 8 patients with recurrent bladder cancer in our cohort.

No.	Gender	Ages	Diagnose	T-Stage	Times of THP Perfusion after TURBT	Time of Recurrence
1	Female	65	NMIBC	Ta	15	18 months
2	Female	60	NMIBC	Ta	15	23 months
3	Male	75	NMIBC	T1	16	17 months
4	Male	56	NMIBC	T1	16	14 months
5	Male	62	NMIBC	T1	11	5 months
6	Male	72	NMIBC	T1	17	28 months
7	Male	79	NMIBC	Ta	16	12 months
8	Male	54	NMIBC	T1	16	27 months

**Table 2:** Clinical information of tissue microarray.

Characteristic		Total Number (N = 70)	
		Numbers (n)	%
Ages	<60 y	30	42.86%
	≥60 y	40	57.14%
Gender	male	60	85.71%
	female	10	14.29%
Tumor size (shortest length)	≤3.0 cm	35	50.00%
	>3.0 cm	15	21.43%
	Unable to calculate/not recorded	20	28.57%
T-stage	Ta	23	32.86%
	T1	27	38.57%
	T2	12	17.14%
	T3	8	11.43%
N-stage	N0	70	100%
M-stage	M0	70	100%
Clinical stage	0	23	32.86%
	Ⅰ	27	38.57%
	Ⅱ	12	17.14%
	Ⅲ	8	11.43%

**Table 3:** Acquisition sequences of AKR1C1.

Prime	Sequence (5′-3′)
AKR1C1(31372-1)-P1	GAGGATCCCCGGGTACCGGTCGCCACCATGGATTCGAAATATCAGTGTG
AKR1C1(31372-1)-P2	TCCTTGTAGTCCATACCATATTCATCAGAAAATGGATAATTAG

**Table 4:** Tumor tissue volume of three groups of mice (cm^3^).

Group/No.	1	2	3	4	5	6	7
Blank (T24)	0.056	0.179	0.381	0.065	0.069	0.287	0.069
Negative Control (T24-CON)	0.381	0.073	0.522	0.069	0.065	0.267	0.065
Overexpression (T24-AKR1C1)	1.879	1.469	1.469	1.469	2.044	0.902	0.902

**Table 5:** Tumor tissue mass of three groups of mice (gram).

Group/No.	1	2	3	4	5	6	7
Blank (T24)	0.08	0.19	0.24	0.08	0.1	0.19	0.1
Negative Control (T24-CON)	0.25	0.14	0.33	0.13	0.09	0.09	0.18
Overexpression (T24-AKR1C1)	0.44	0.39	0.38	0.36	0.46	0.22	0.23

## Data Availability

All the original data involved in this study can be requested from the first author (Z.N.) or corresponding author (S.Z.). We promise to make all original data in this study available to readers for free.

## Supplementary Materials

The following supporting information can be downloaded at: https://www.mdpi.com/article/10.3390/cancers15092487/s1, Figures S1–S33: Supplementary Information.
