# Supplementary figures and images for: Genome-Wide Screening Identifies Gene AKR1C1 Critical for Resistance to Pirarubicin in Bladder Cancer
Source: Cancers (Basel). 2023 Apr 26;15(9):2487. doi: 10.3390/cancers15092487 (PMC10177075; doi:10.3390/cancers15092487)

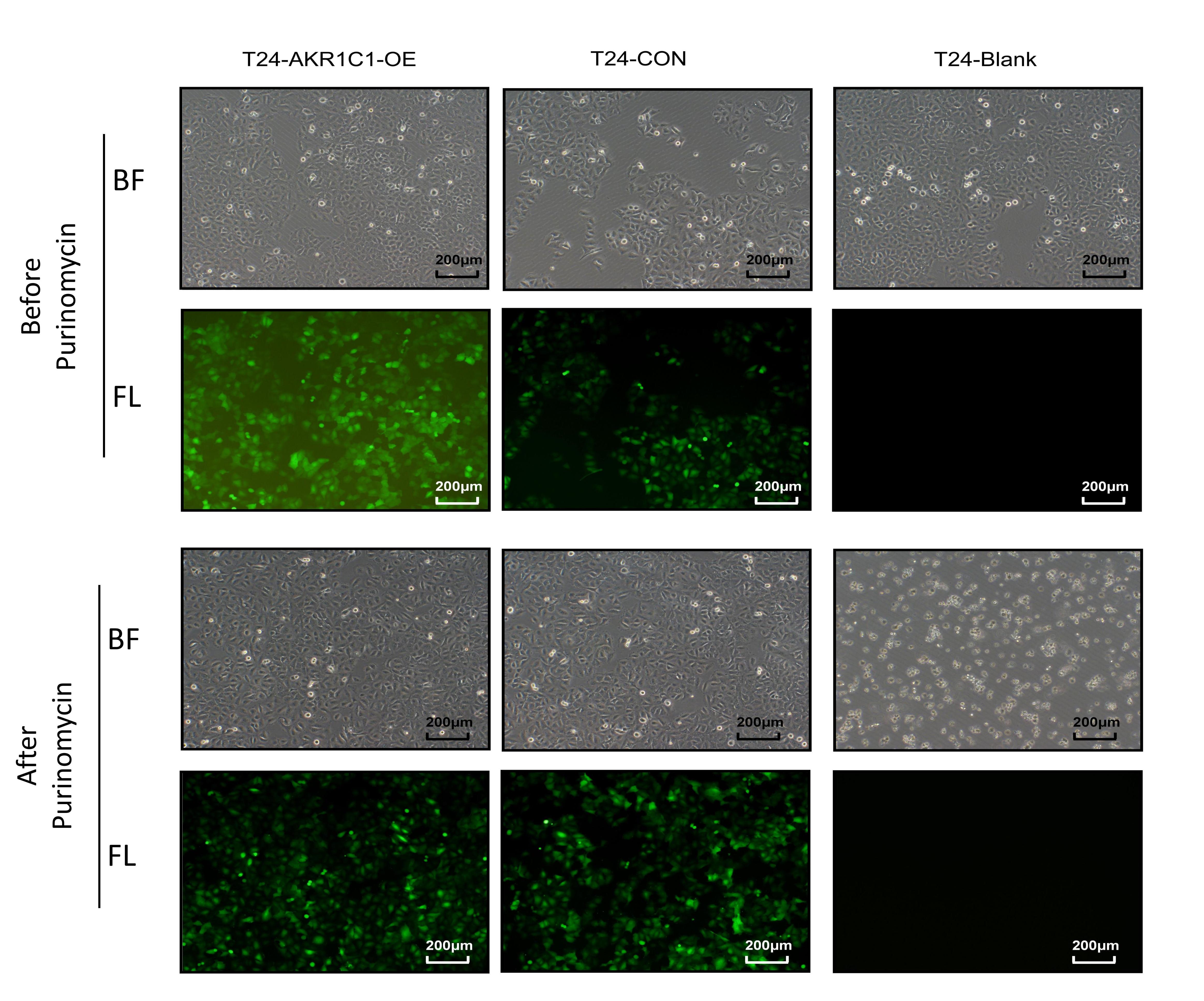

Supplement: Supplementary file 1 [file cancers-15-02487-s001.zip › Figure S1.jpg]

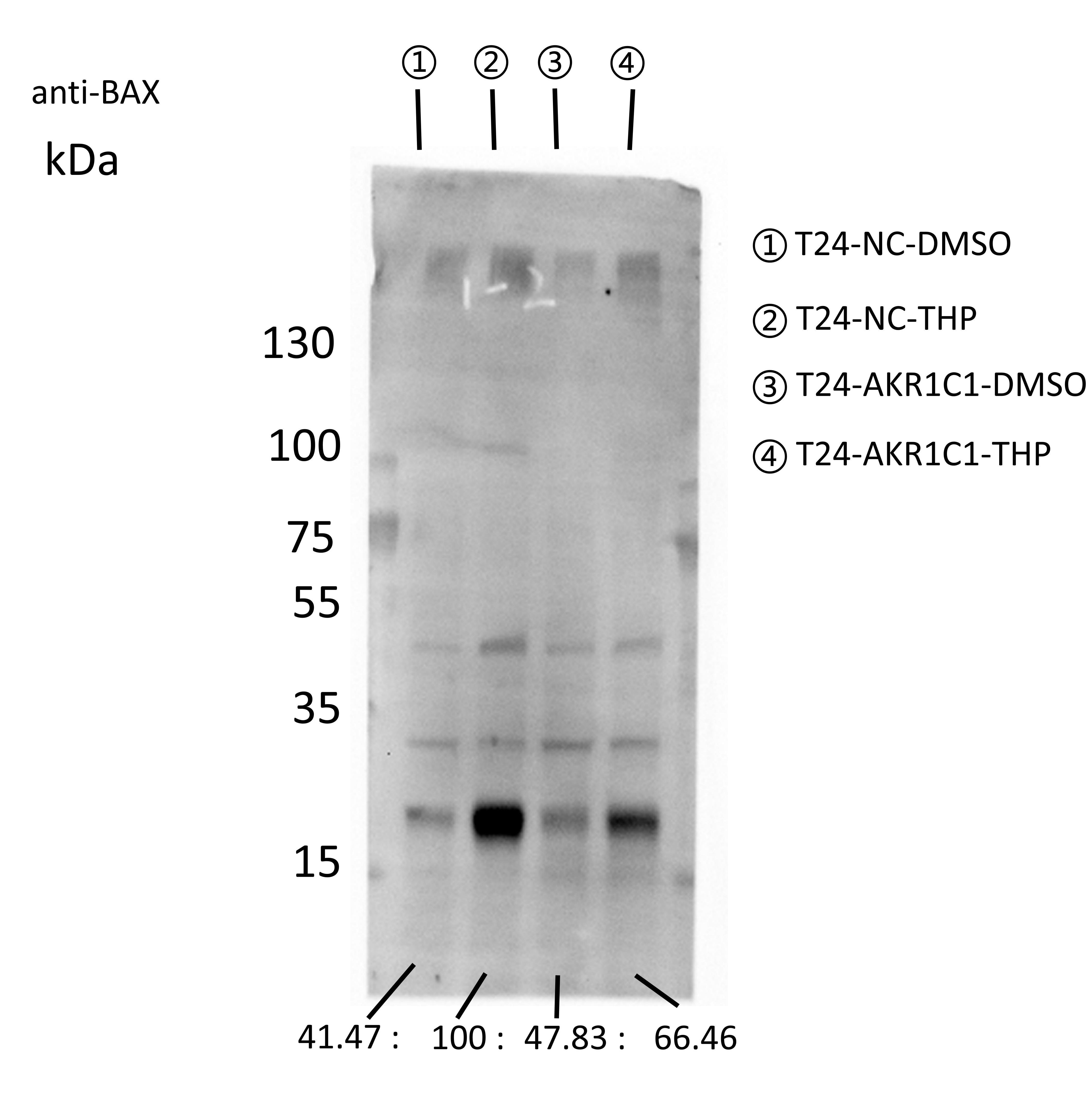

Supplement: Supplementary file 1 [file cancers-15-02487-s001.zip › Figure S10_BAX.jpg]

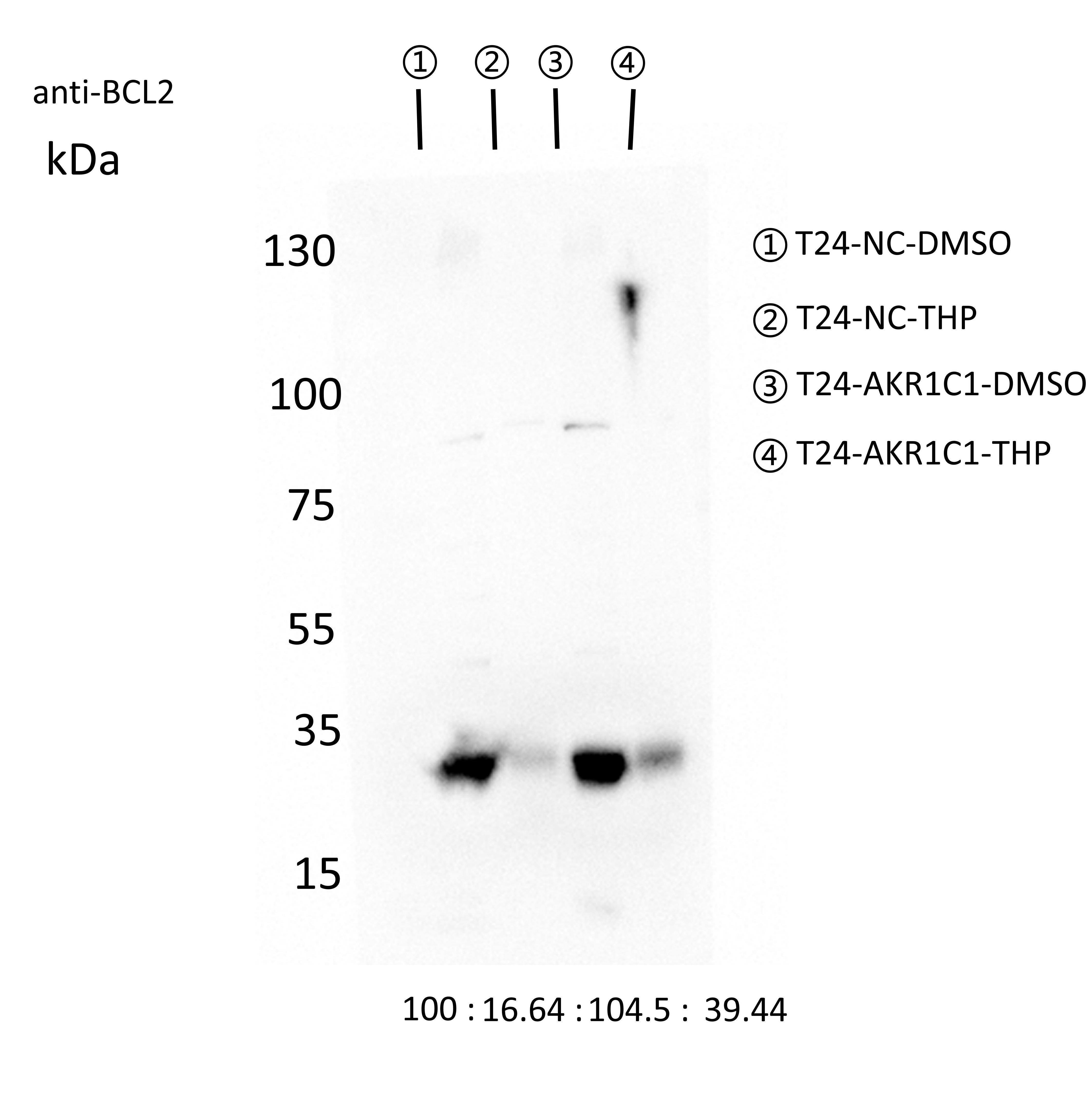

Supplement: Supplementary file 1 [file cancers-15-02487-s001.zip › Figure S11_BCL2.jpg]

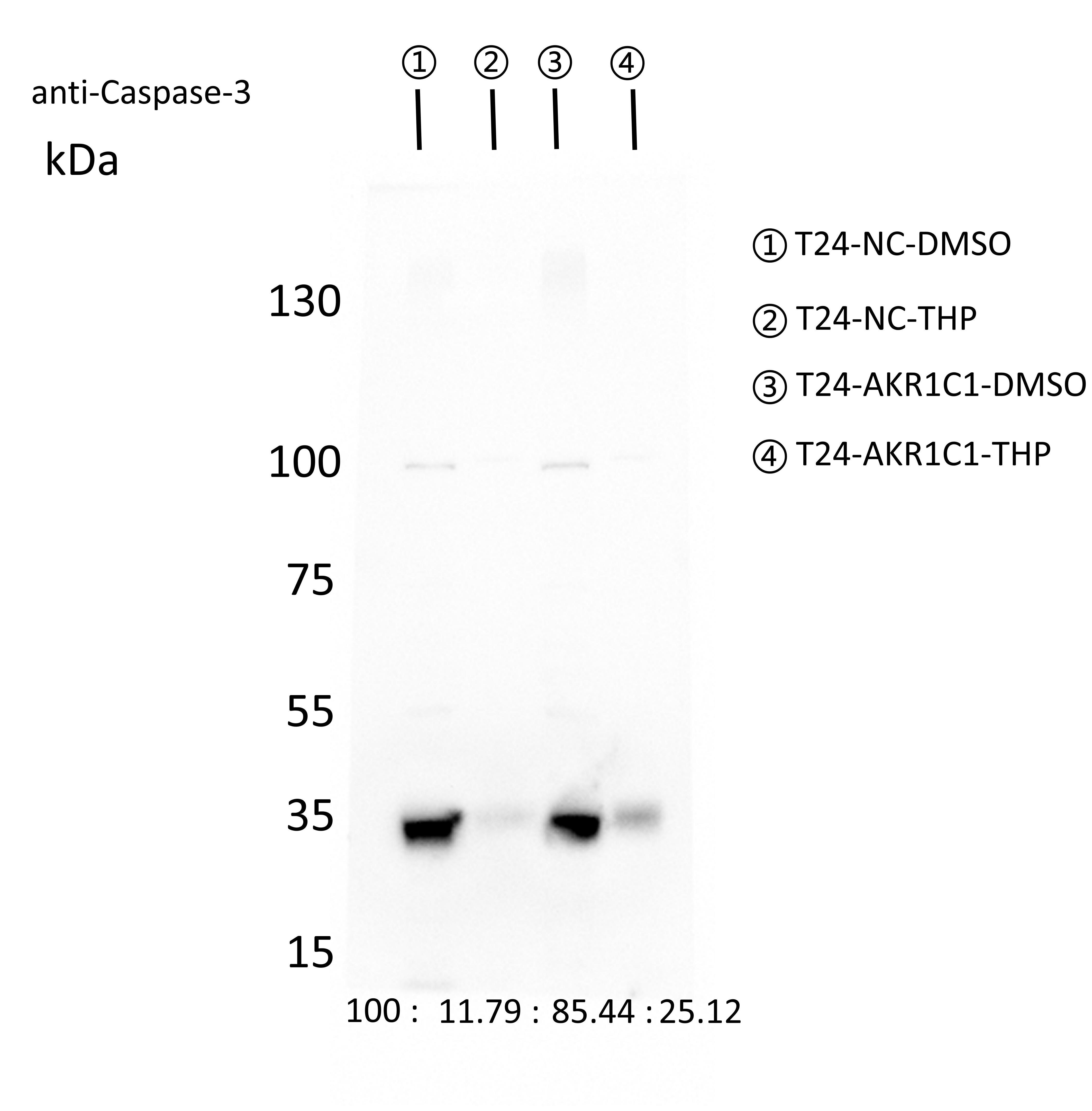

Supplement: Supplementary file 1 [file cancers-15-02487-s001.zip › Figure S12_Caspase 3.jpg]

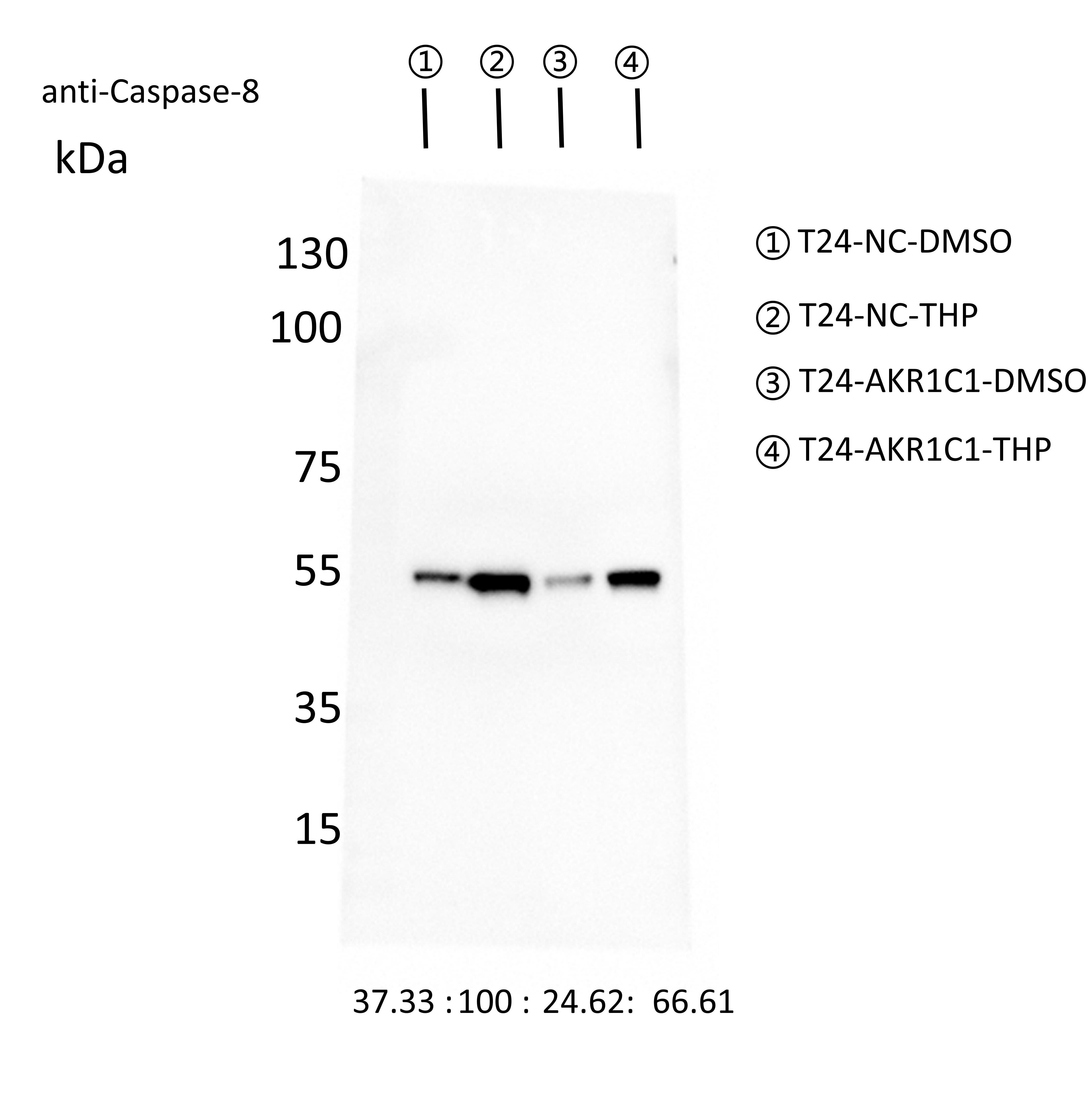

Supplement: Supplementary file 1 [file cancers-15-02487-s001.zip › Figure S13_Caspase 8.jpg]

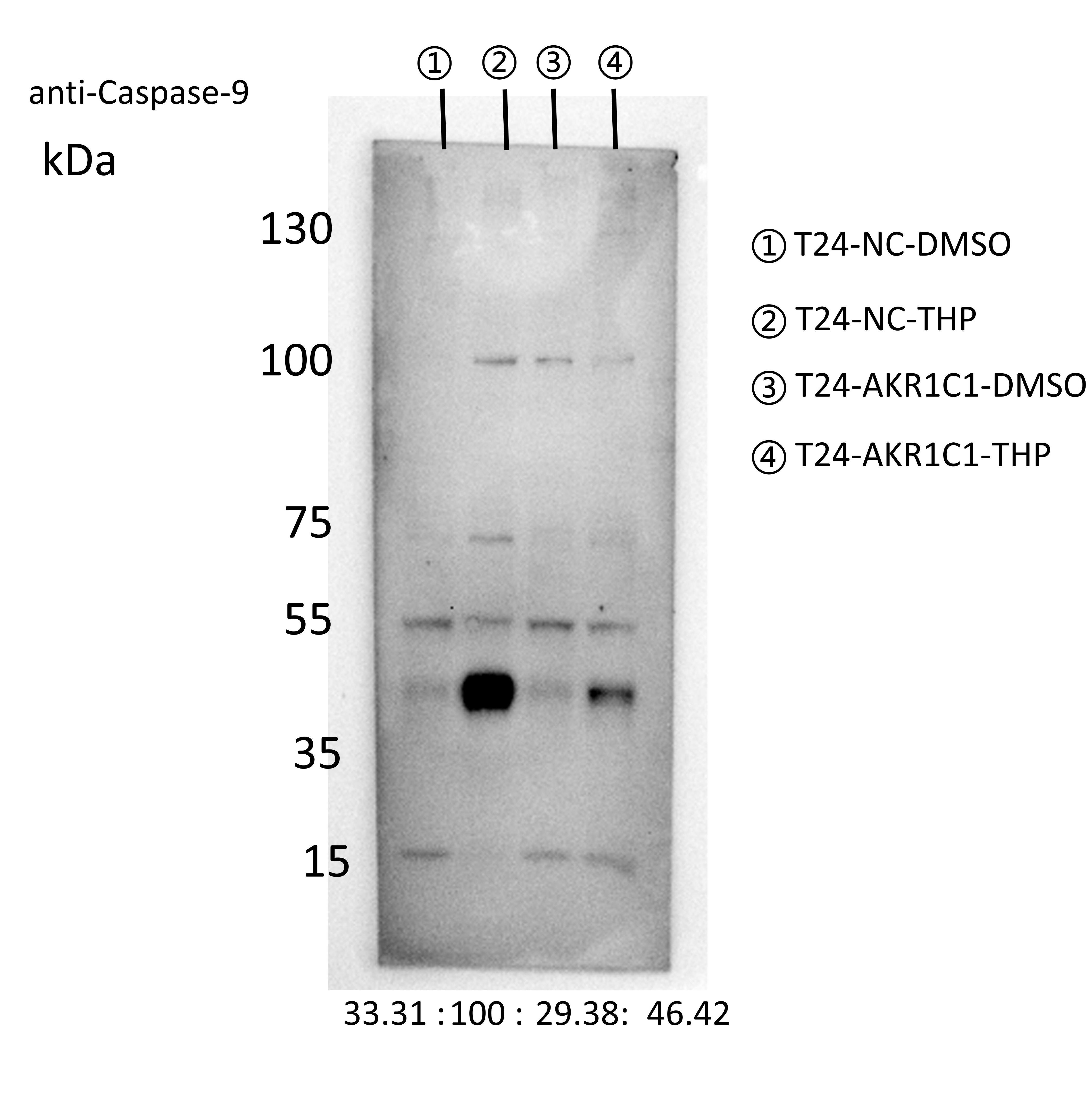

Supplement: Supplementary file 1 [file cancers-15-02487-s001.zip › Figure S14_Caspase 9.jpg]

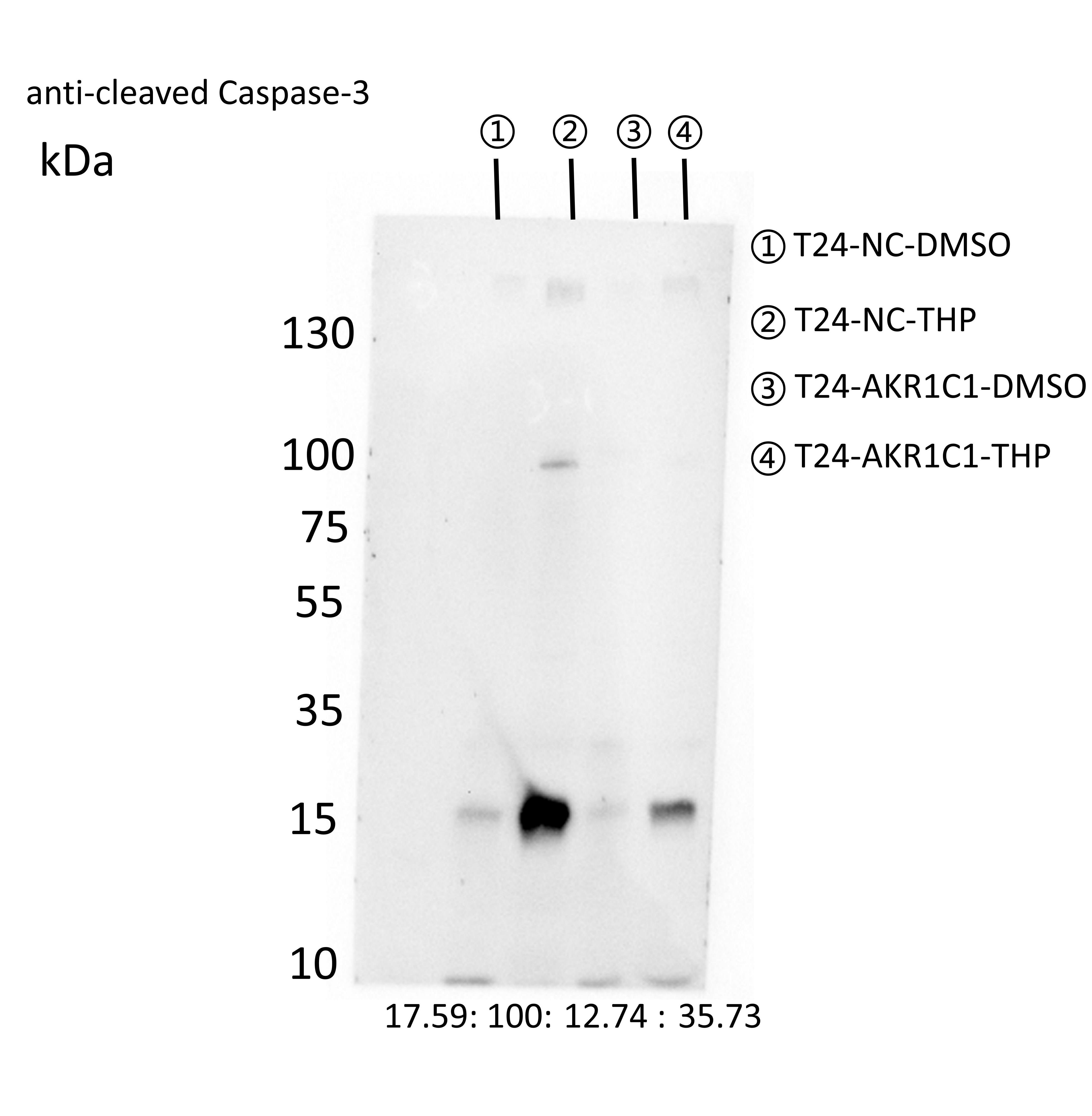

Supplement: Supplementary file 1 [file cancers-15-02487-s001.zip › Figure S15_cleaved Caspase 3.jpg]

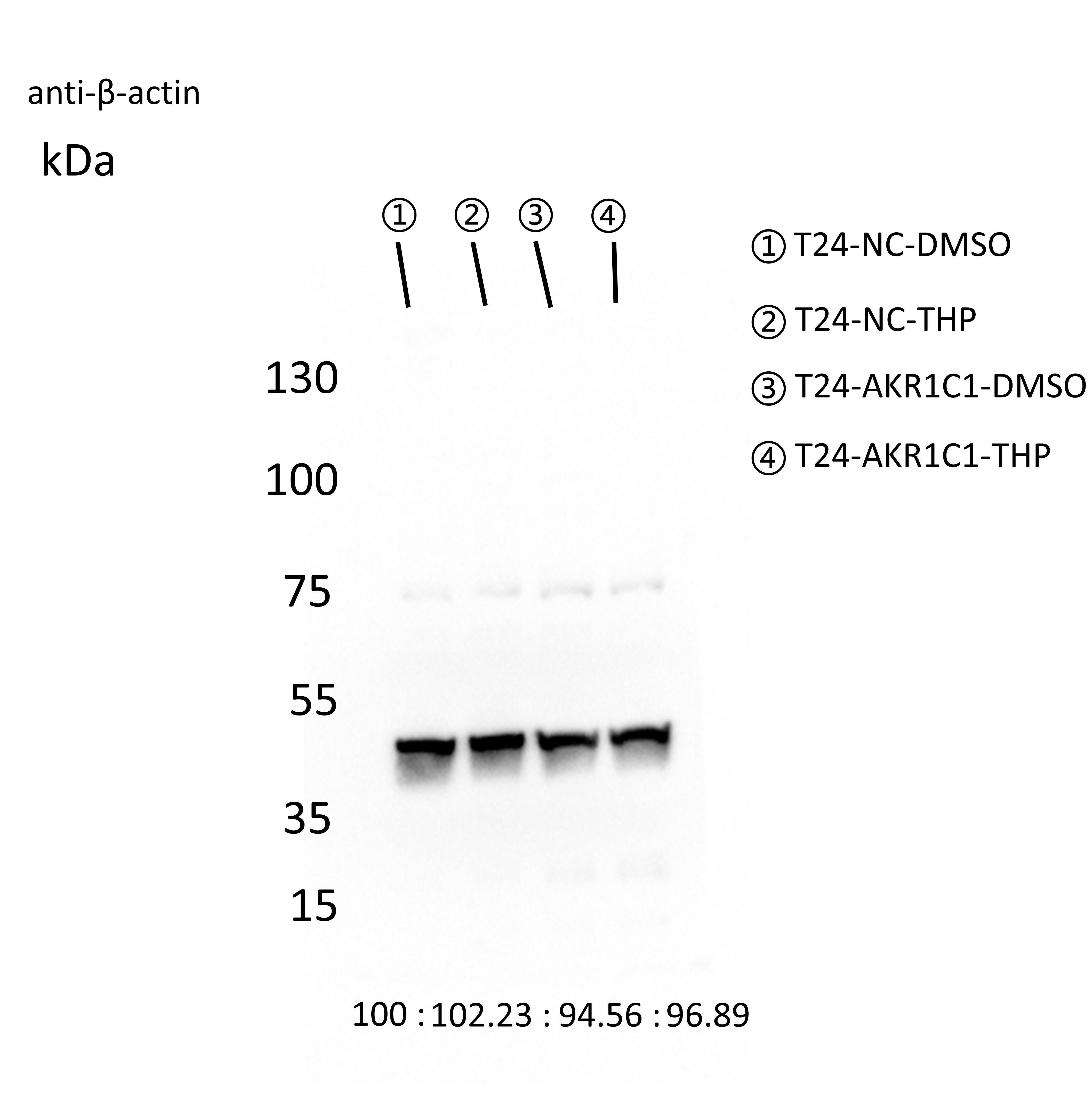

Supplement: Supplementary file 1 [file cancers-15-02487-s001.zip › Figure S16_a┬-actin.jpg]

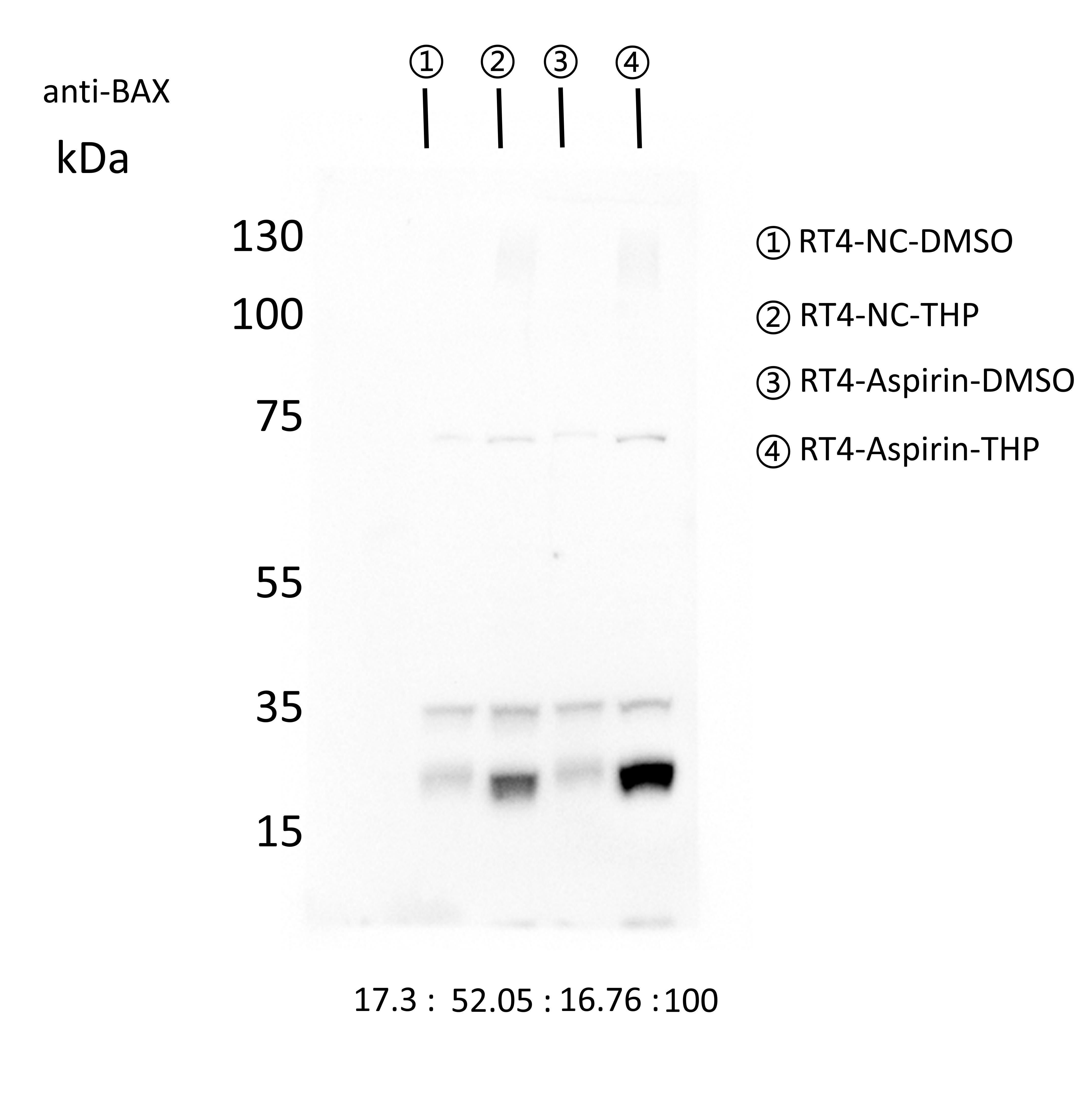

Supplement: Supplementary file 1 [file cancers-15-02487-s001.zip › Figure S17_BAX.jpg]

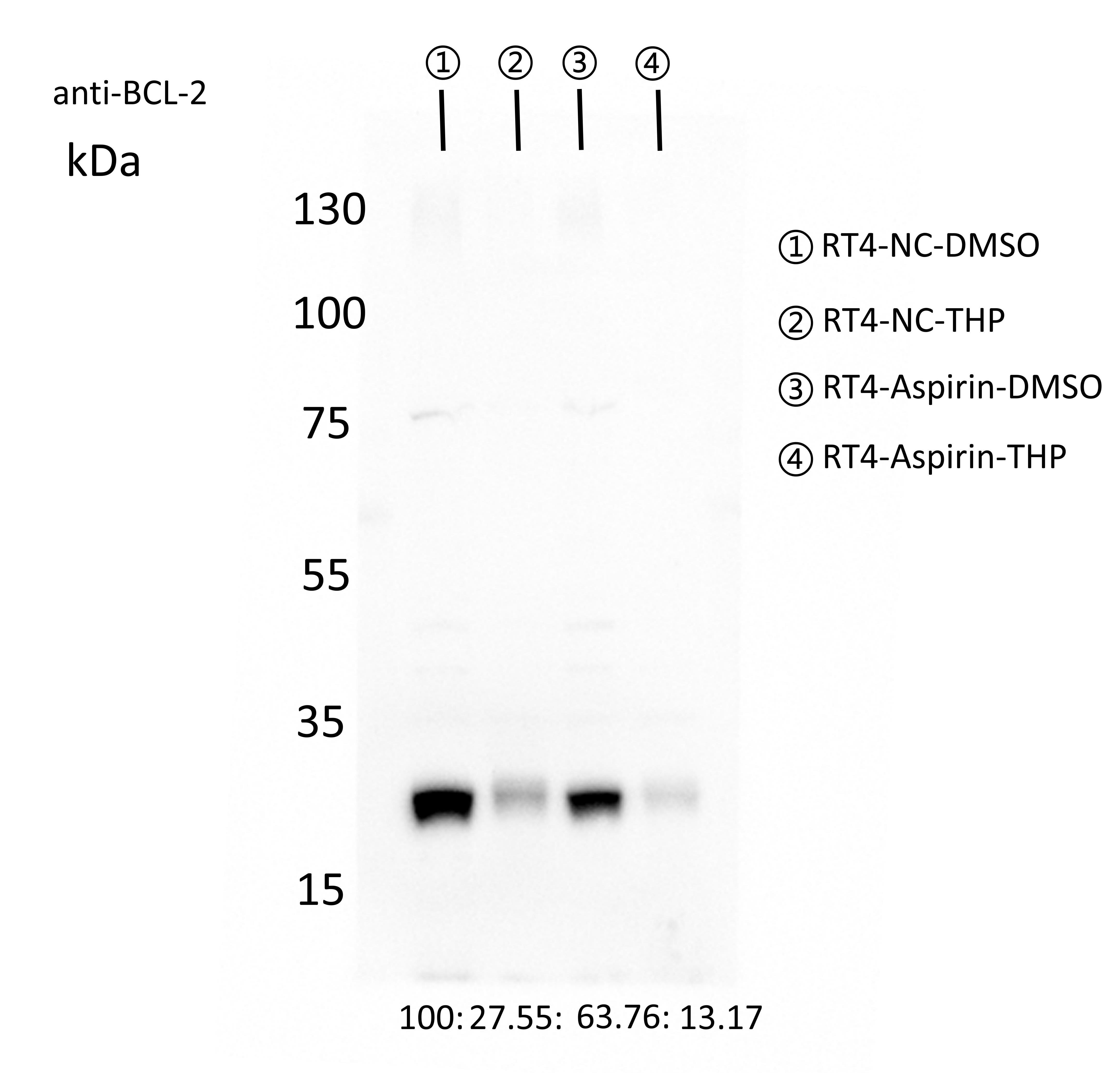

Supplement: Supplementary file 1 [file cancers-15-02487-s001.zip › Figure S18_BCL2.jpg]

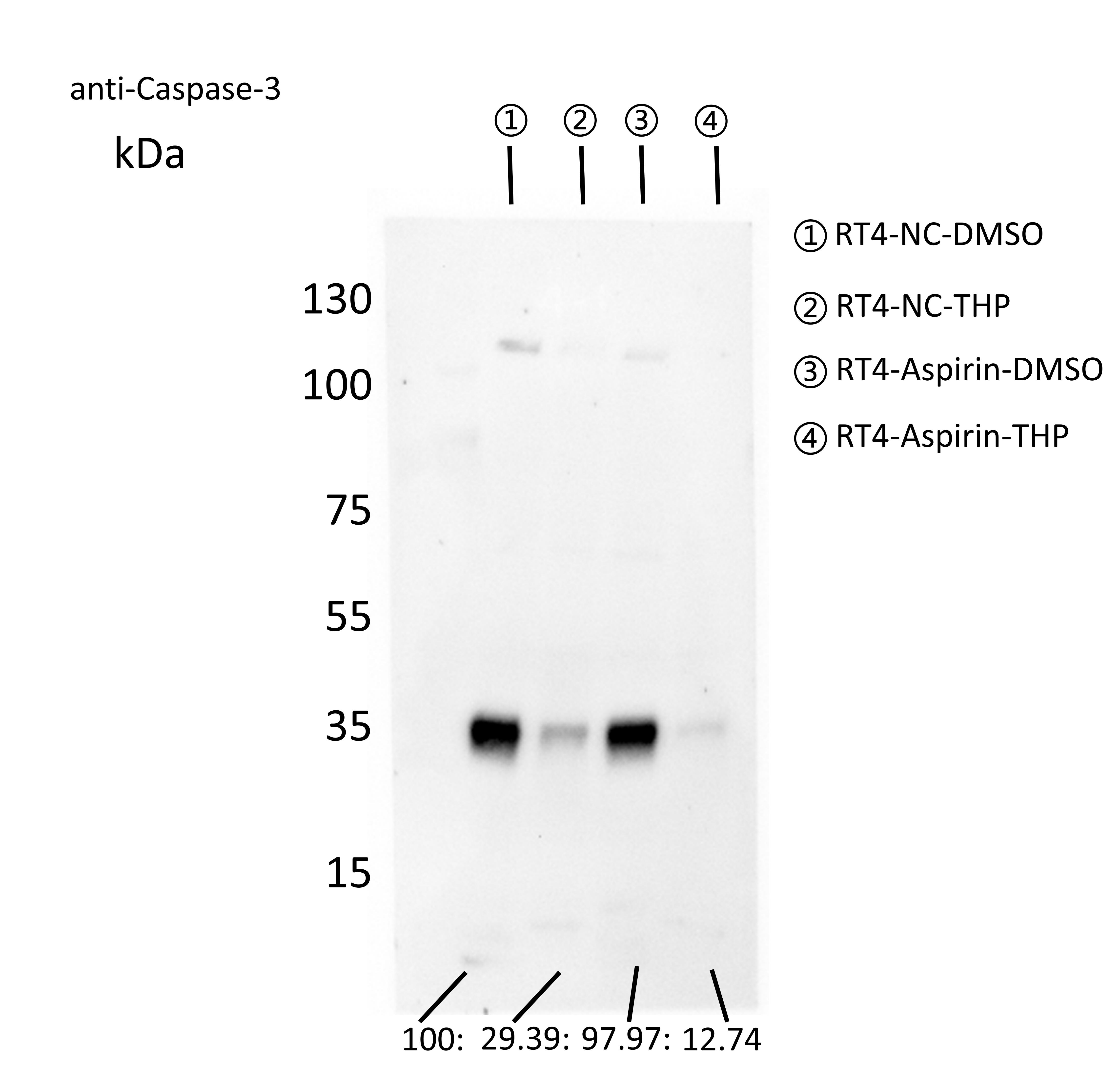

Supplement: Supplementary file 1 [file cancers-15-02487-s001.zip › Figure S19_Caspase 3.jpg]

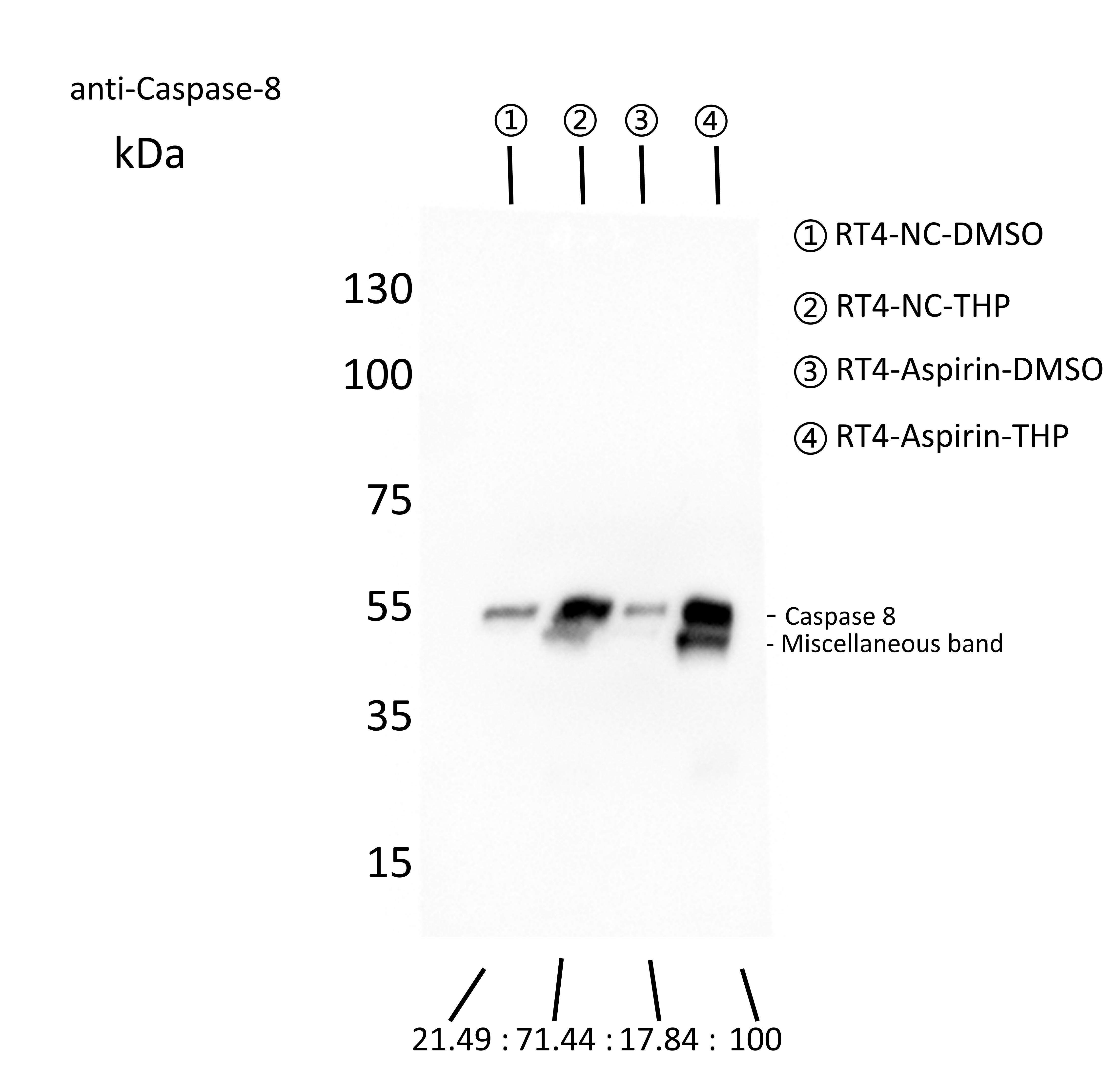

Supplement: Supplementary file 1 [file cancers-15-02487-s001.zip › Figure S20_Caspase 8.jpg]

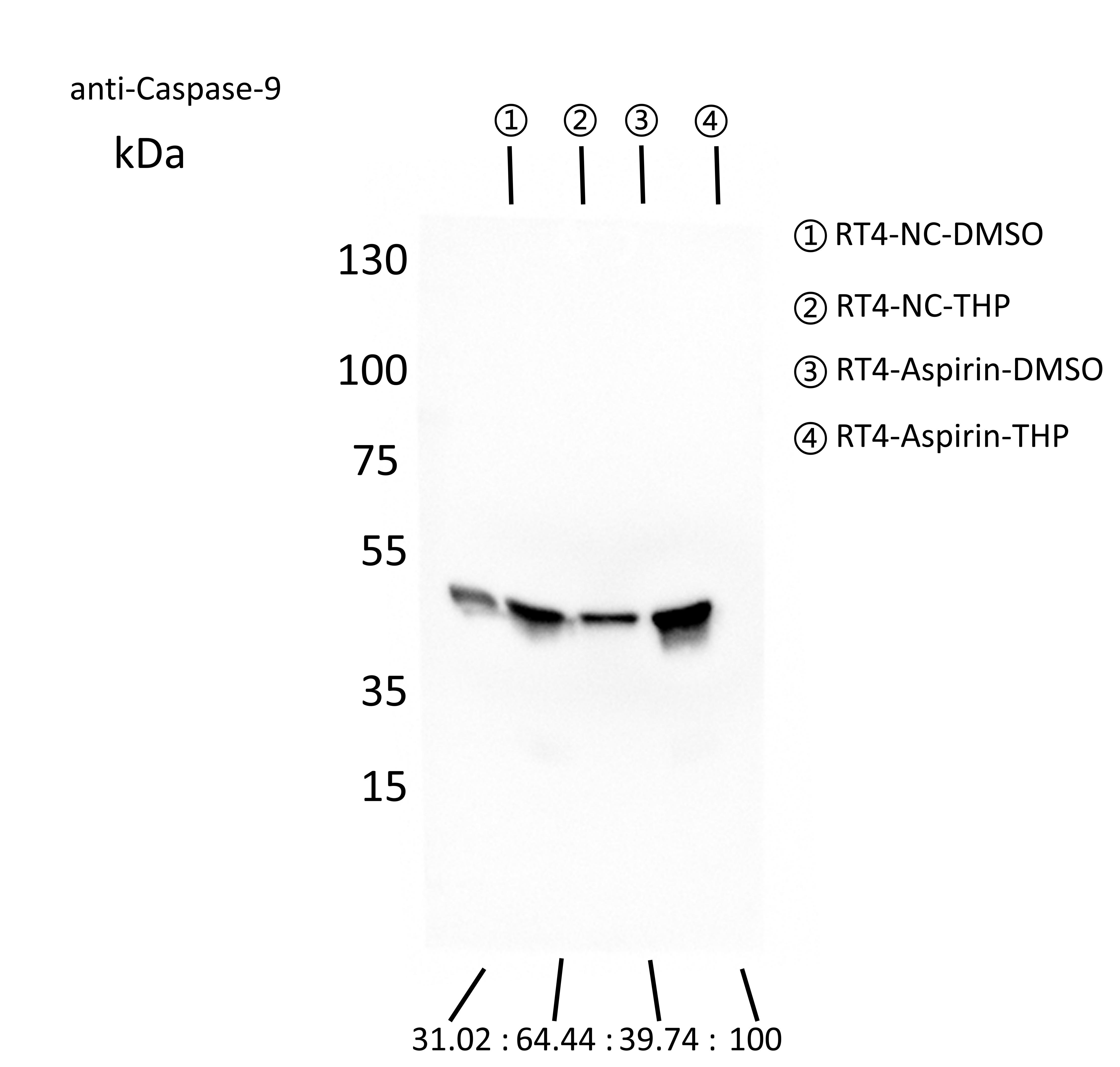

Supplement: Supplementary file 1 [file cancers-15-02487-s001.zip › Figure S21_Caspase 9.jpg]

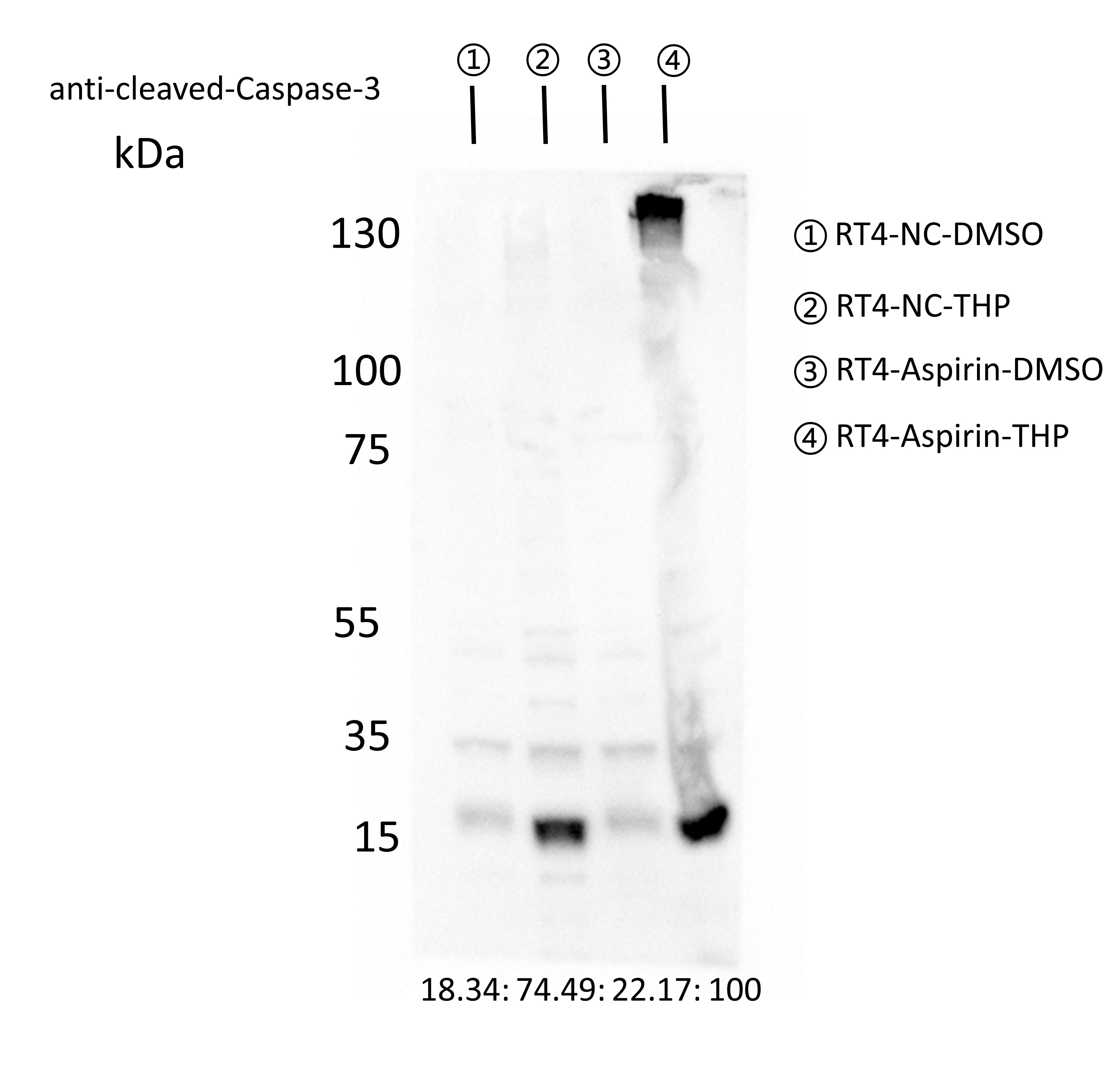

Supplement: Supplementary file 1 [file cancers-15-02487-s001.zip › Figure S22_cleaved Caspase 3.jpg]

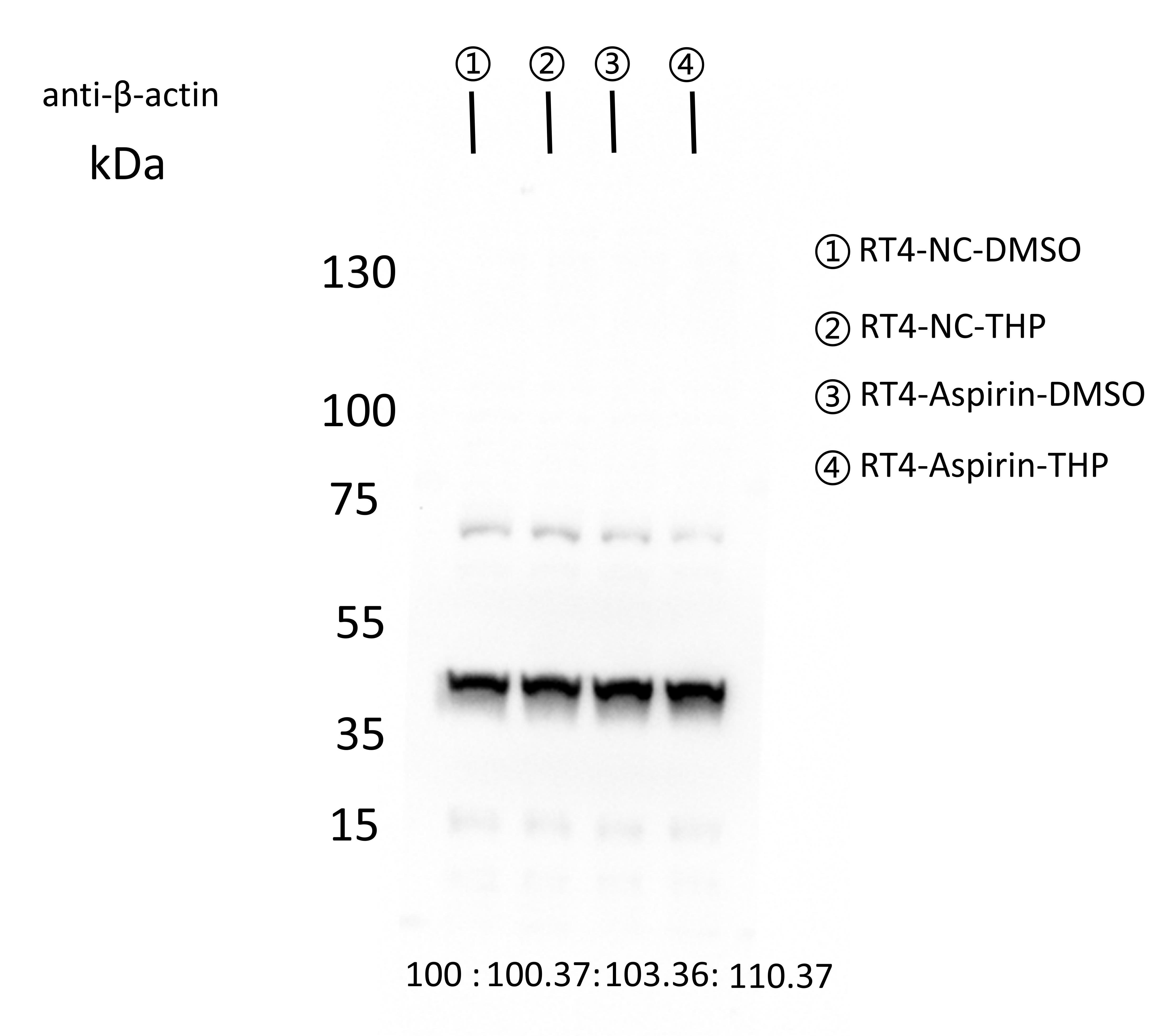

Supplement: Supplementary file 1 [file cancers-15-02487-s001.zip › Figure S23_a┬-actin.jpg]

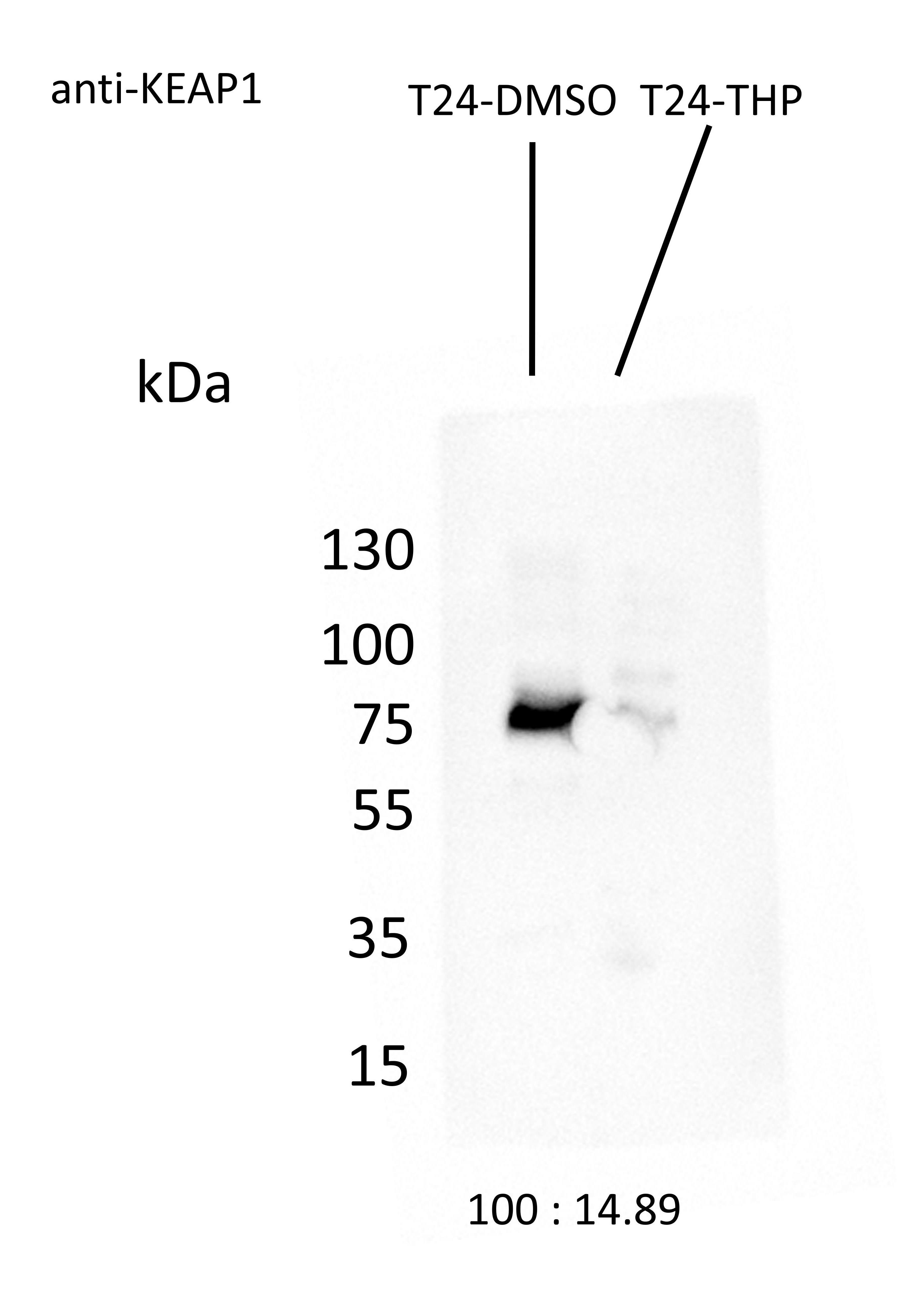

Supplement: Supplementary file 1 [file cancers-15-02487-s001.zip › Figure S24_KEAP1.jpg]

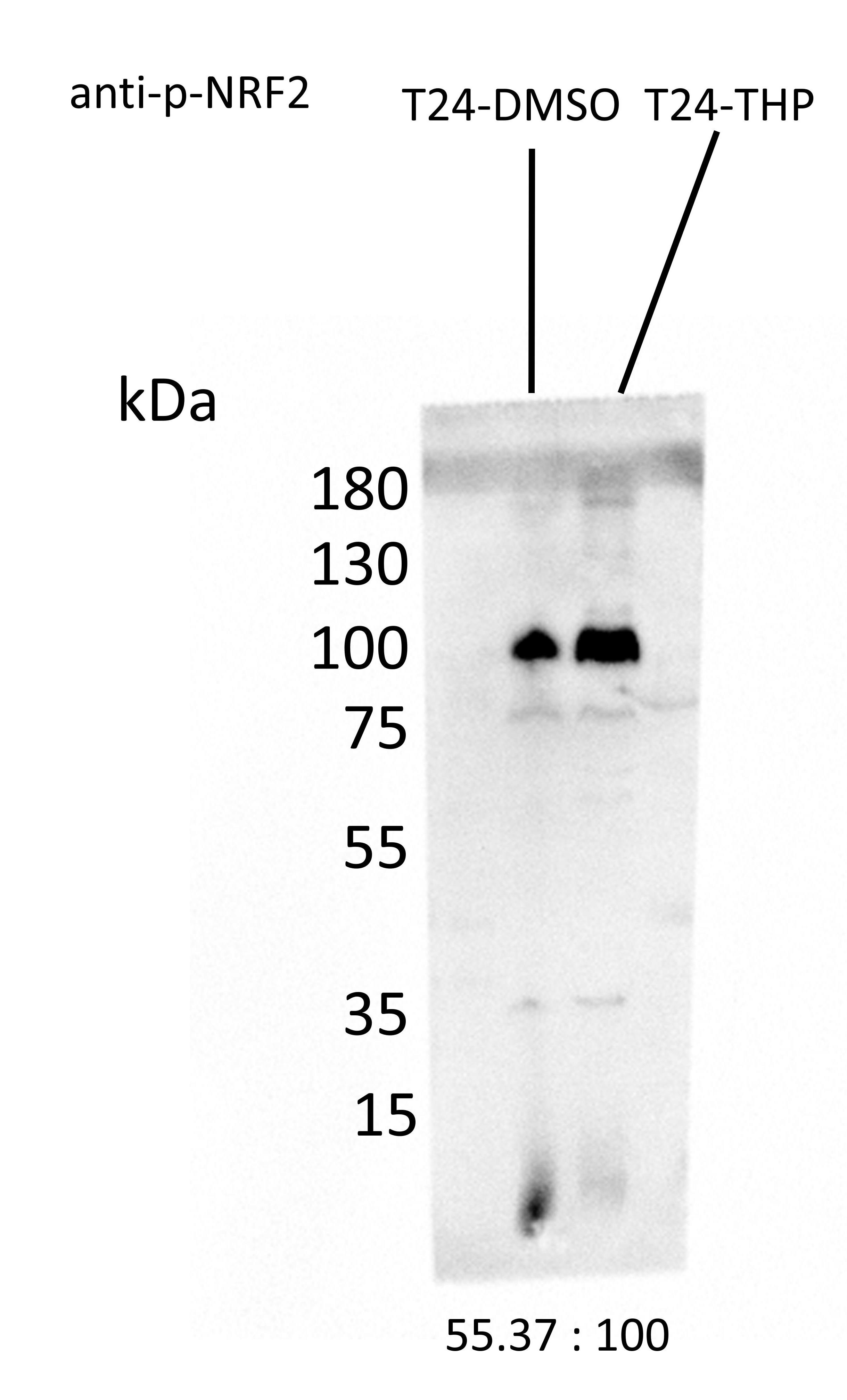

Supplement: Supplementary file 1 [file cancers-15-02487-s001.zip › Figure S25_p-NRF2.jpg]

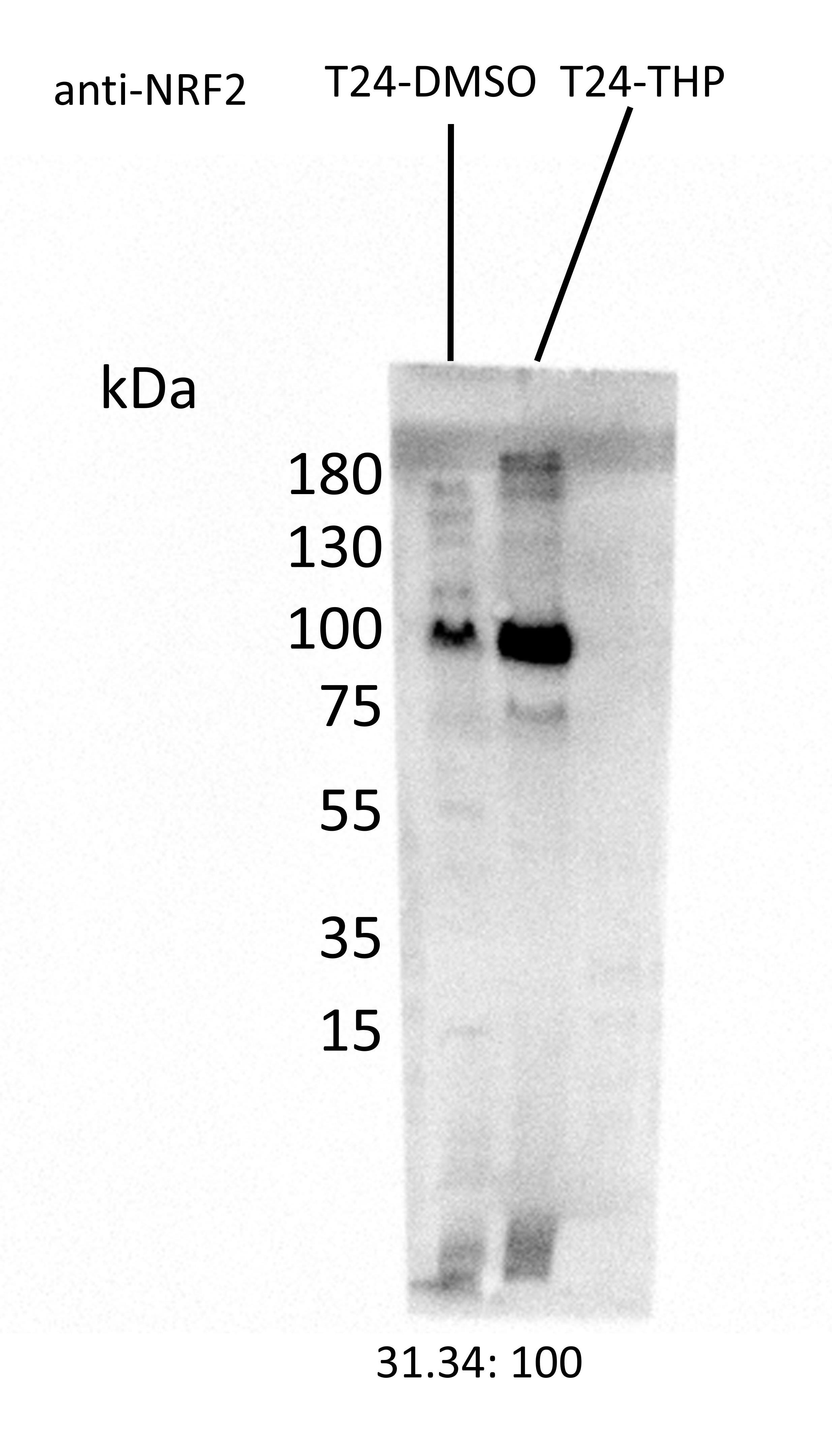

Supplement: Supplementary file 1 [file cancers-15-02487-s001.zip › Figure S26_NRF2.jpg]

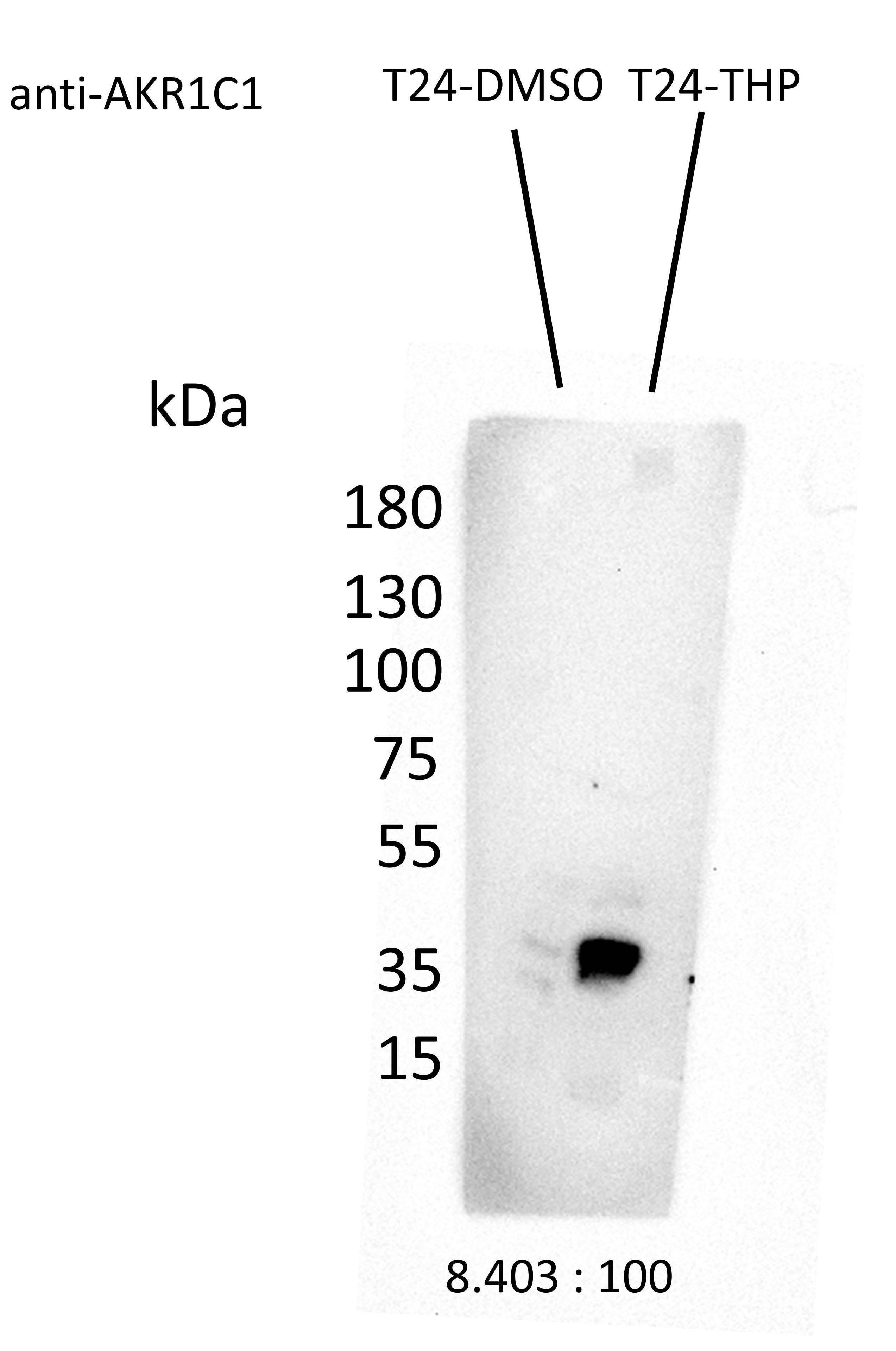

Supplement: Supplementary file 1 [file cancers-15-02487-s001.zip › Figure S27_AKR1C1.jpg]

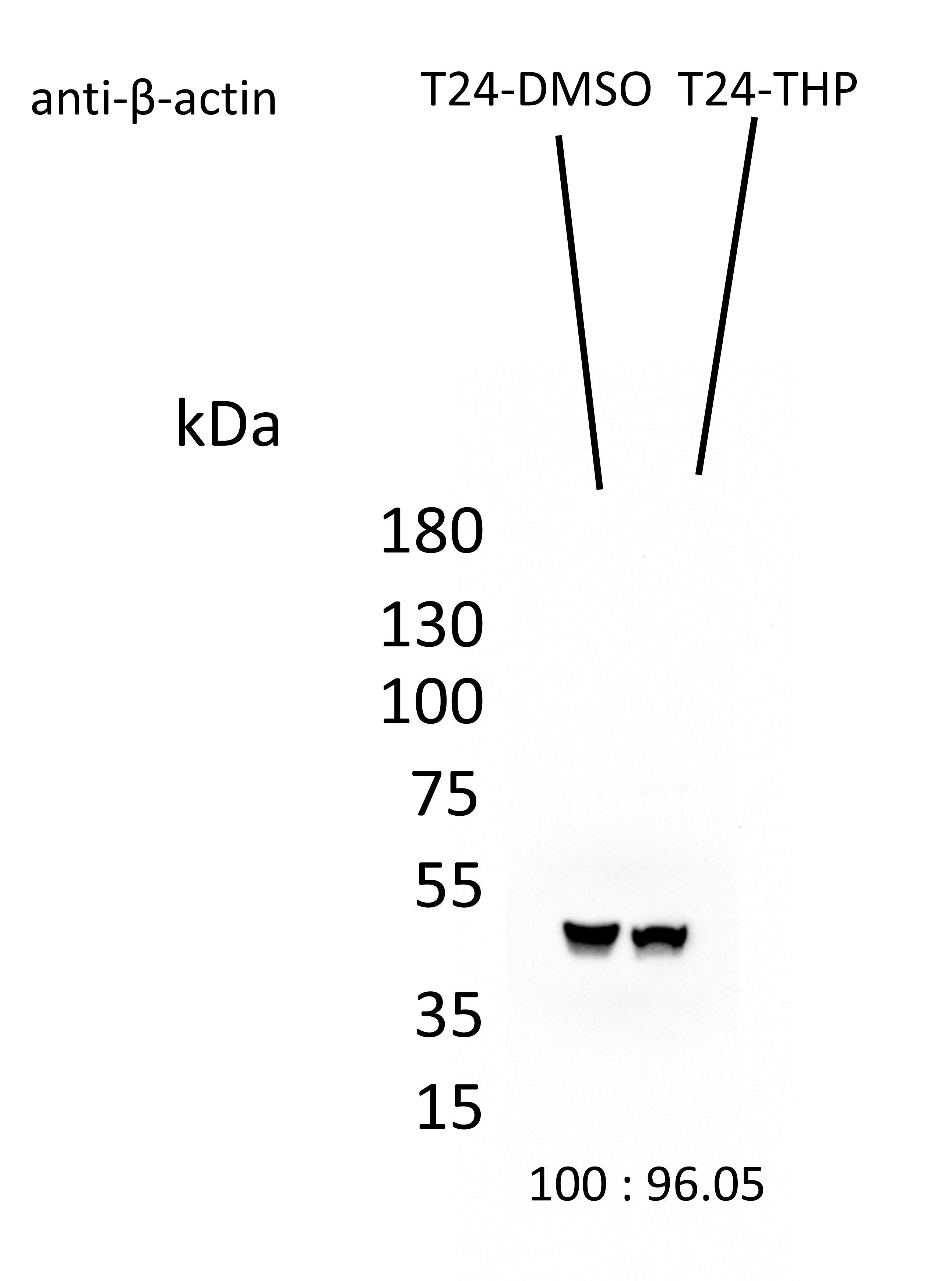

Supplement: Supplementary file 1 [file cancers-15-02487-s001.zip › Figure S28_a┬-actin.jpg]

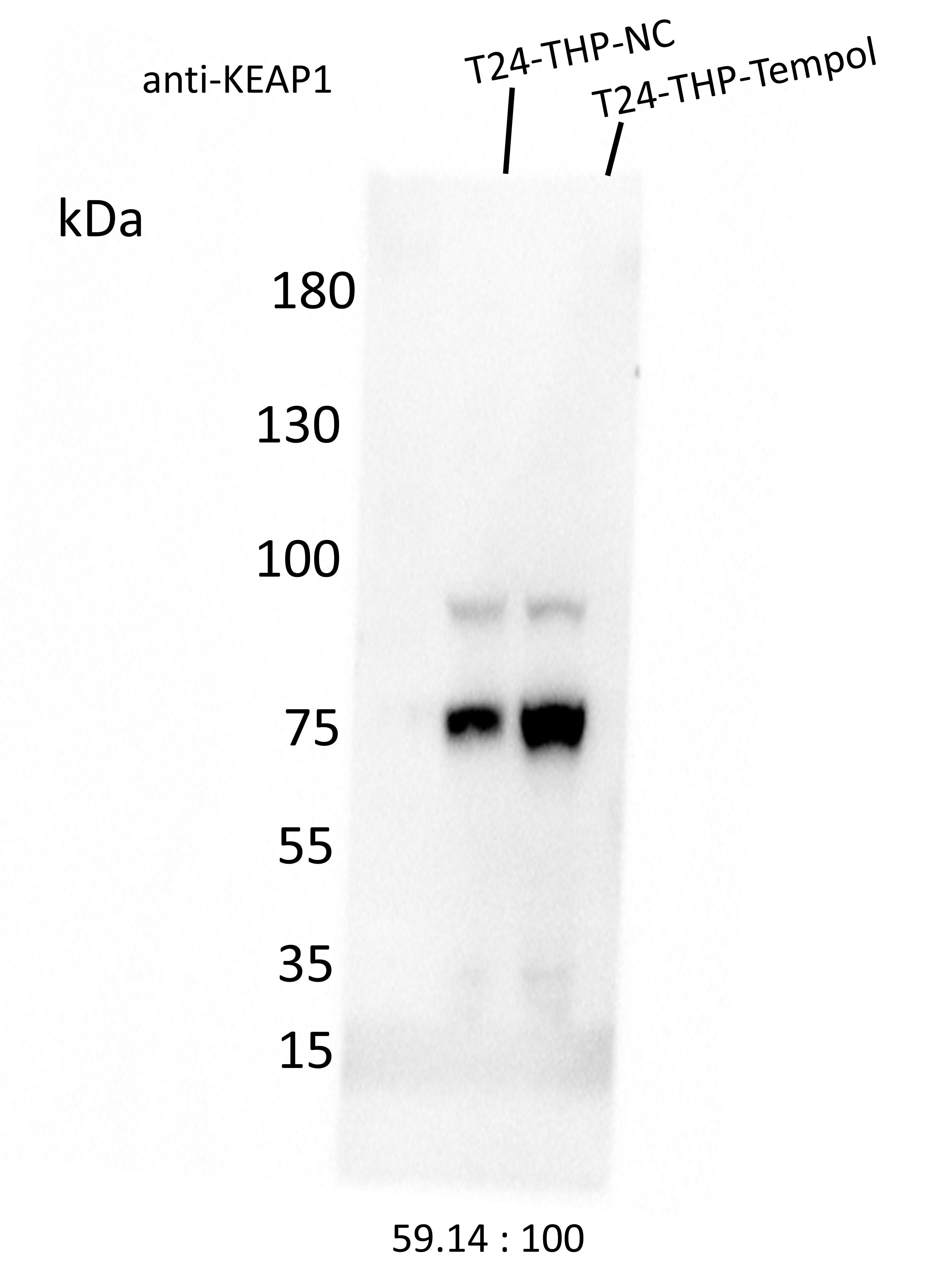

Supplement: Supplementary file 1 [file cancers-15-02487-s001.zip › Figure S29_KEAP1.jpg]

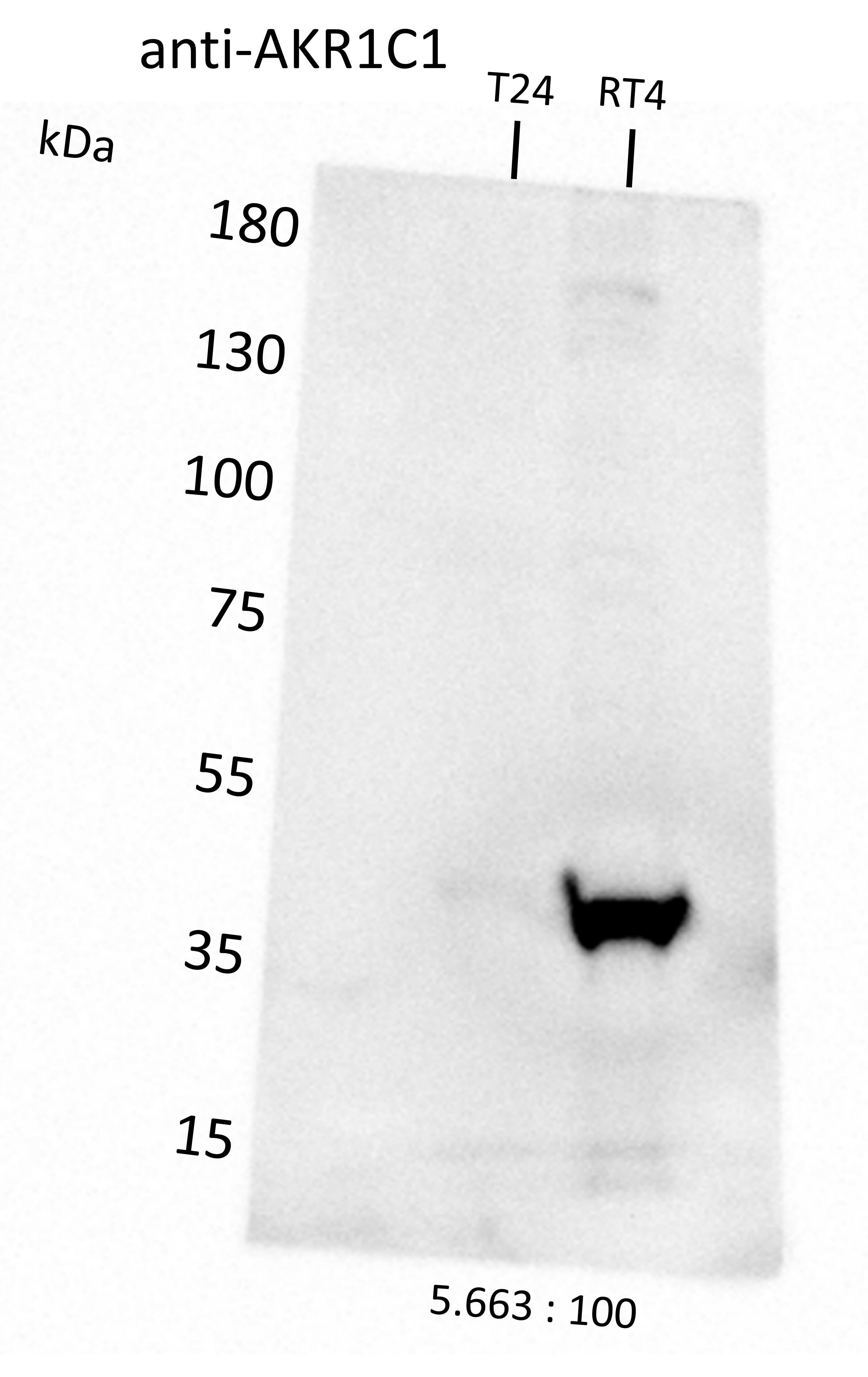

Supplement: Supplementary file 1 [file cancers-15-02487-s001.zip › Figure S2_AKR1C1.jpg]

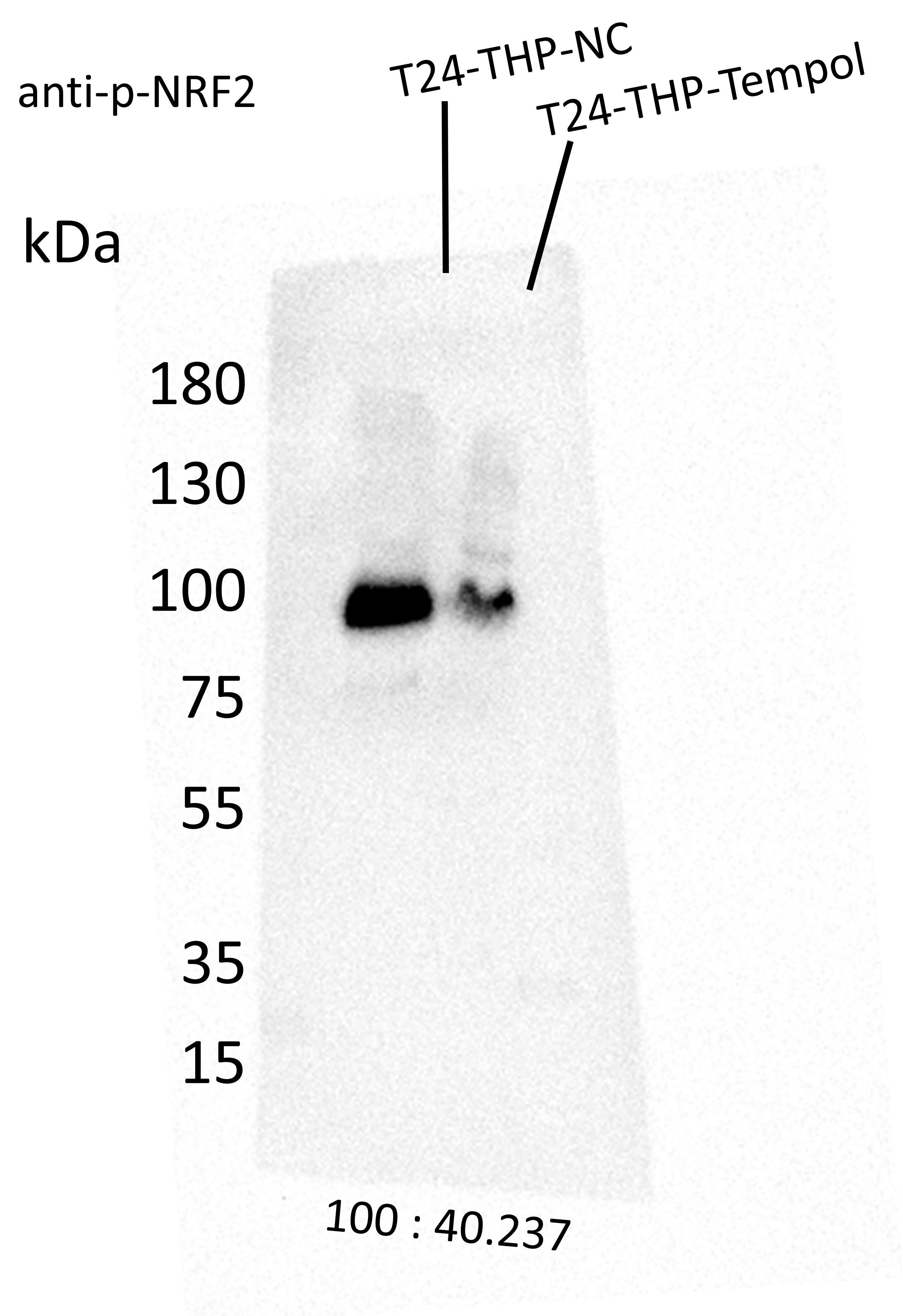

Supplement: Supplementary file 1 [file cancers-15-02487-s001.zip › Figure S30_p-NRF2.jpg]

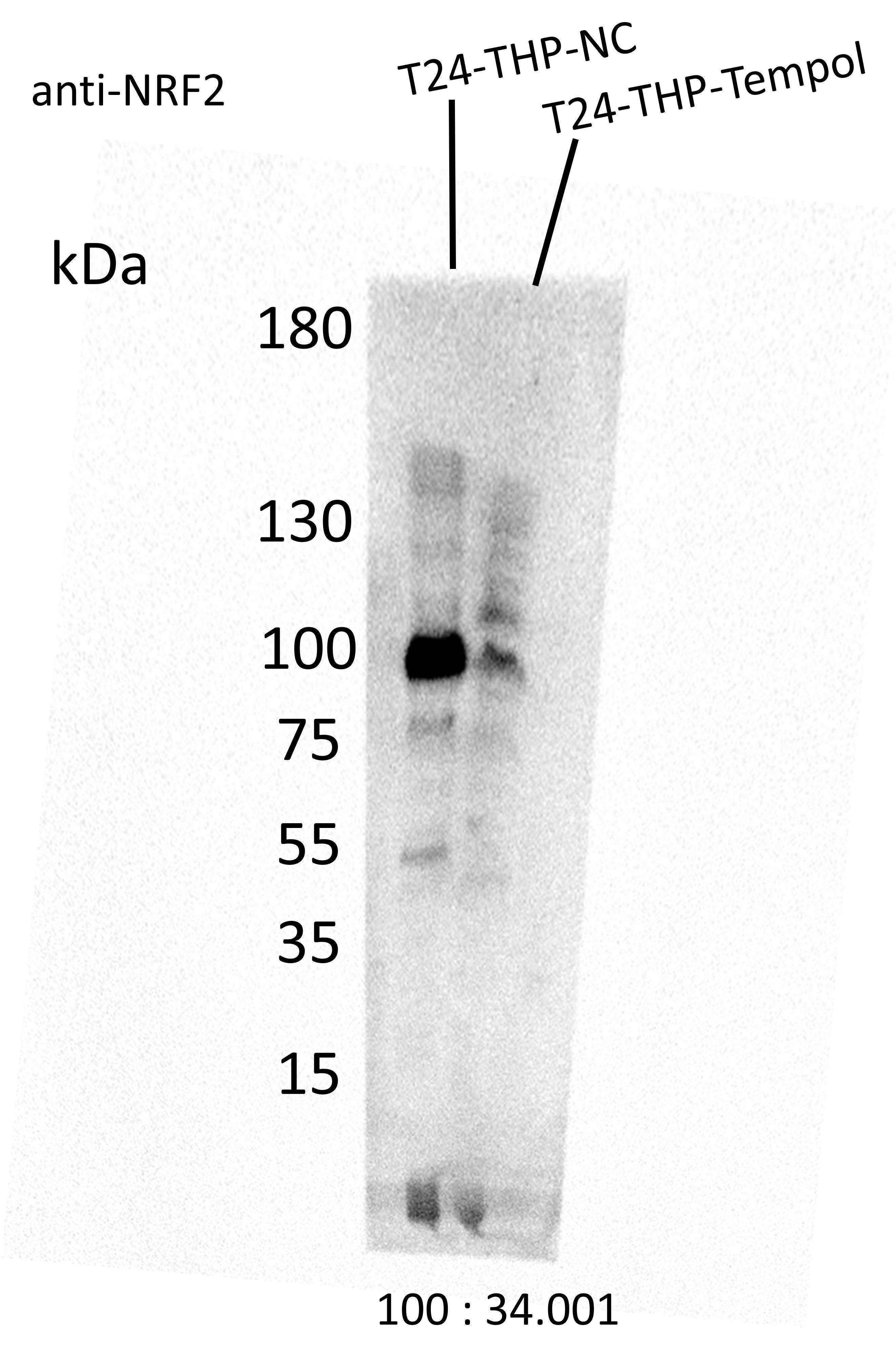

Supplement: Supplementary file 1 [file cancers-15-02487-s001.zip › Figure S31_NRF2.jpg]

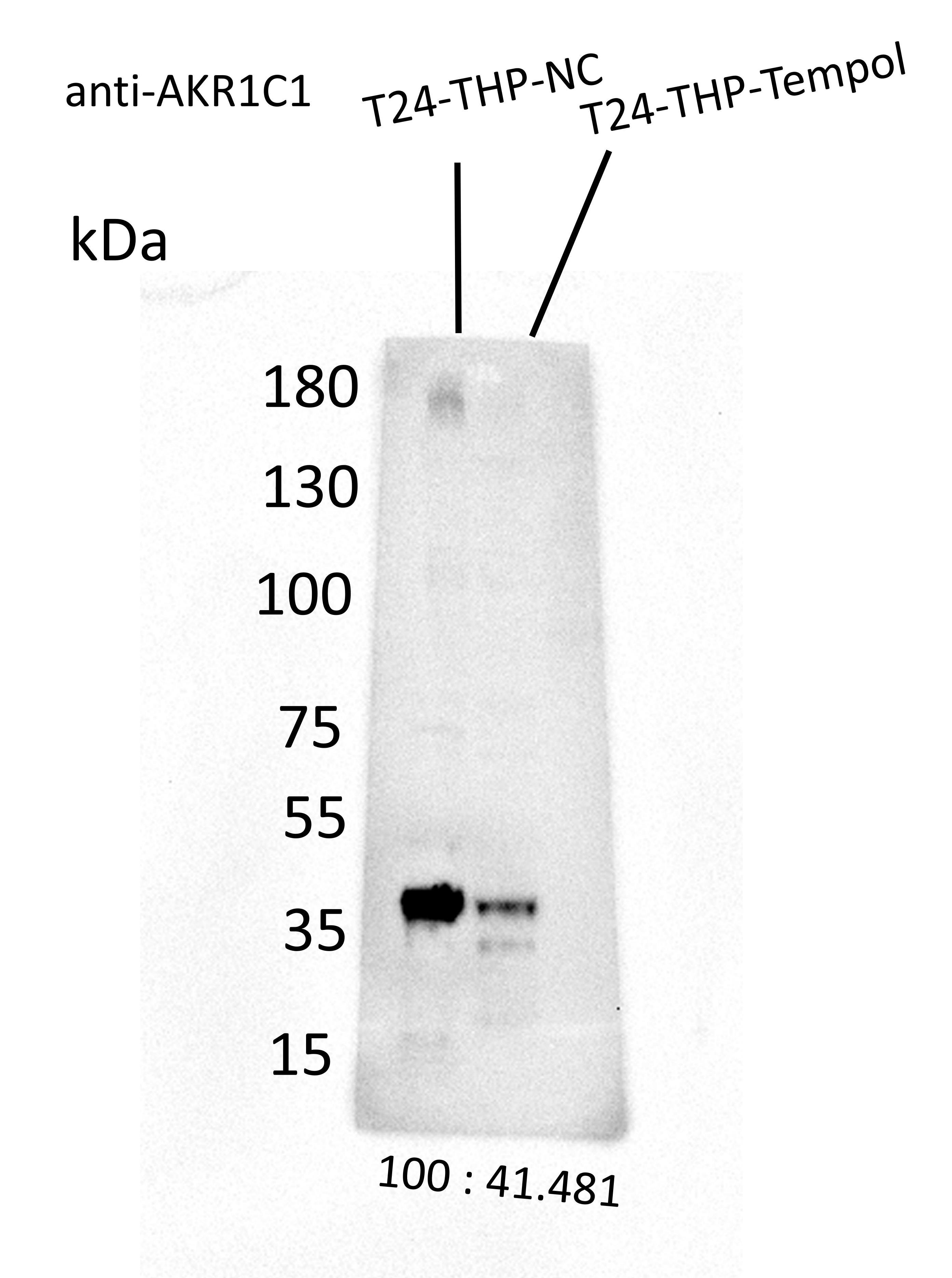

Supplement: Supplementary file 1 [file cancers-15-02487-s001.zip › Figure S32_AKR1C1.jpg]

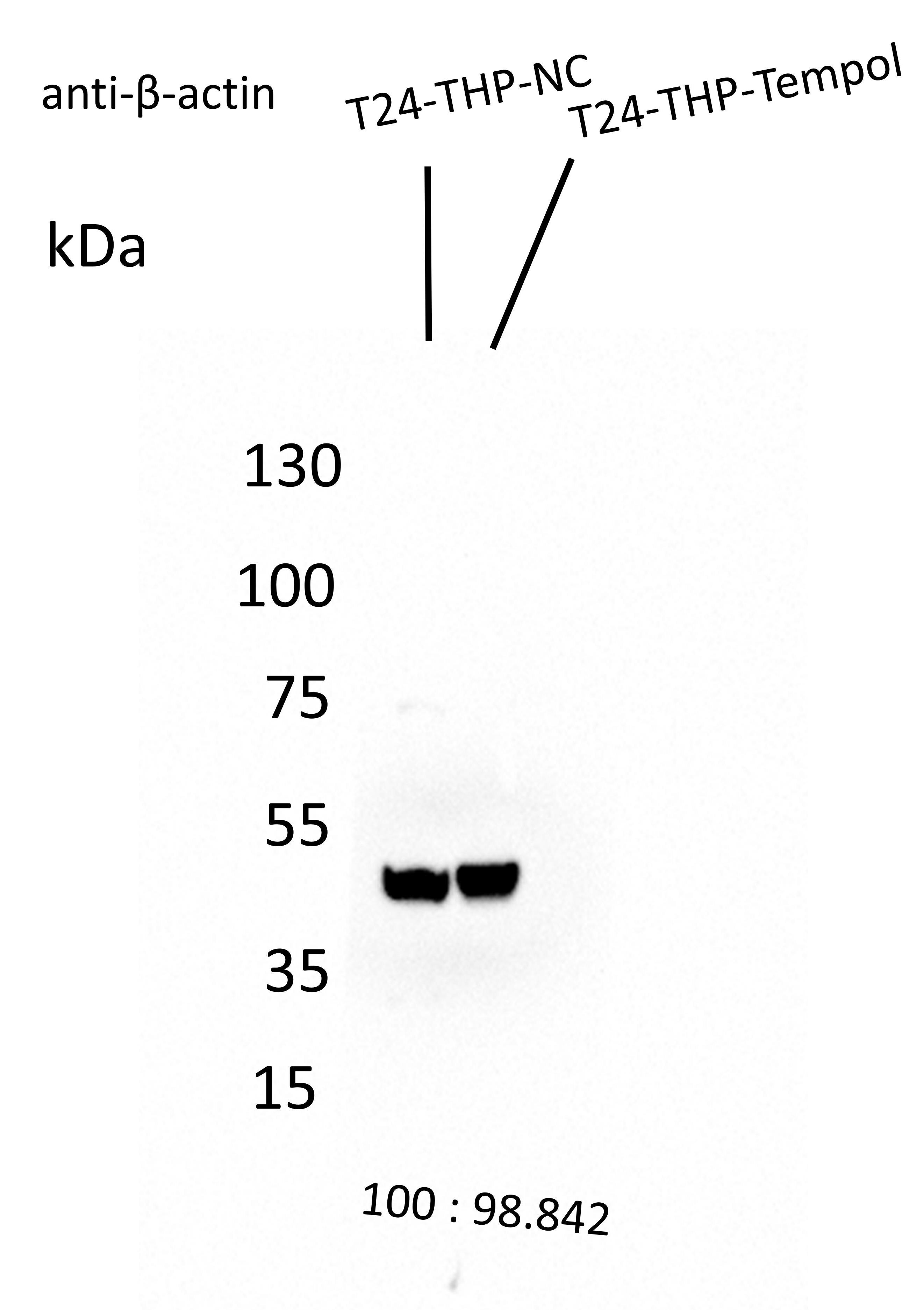

Supplement: Supplementary file 1 [file cancers-15-02487-s001.zip › Figure S33_a┬-actin.jpg]

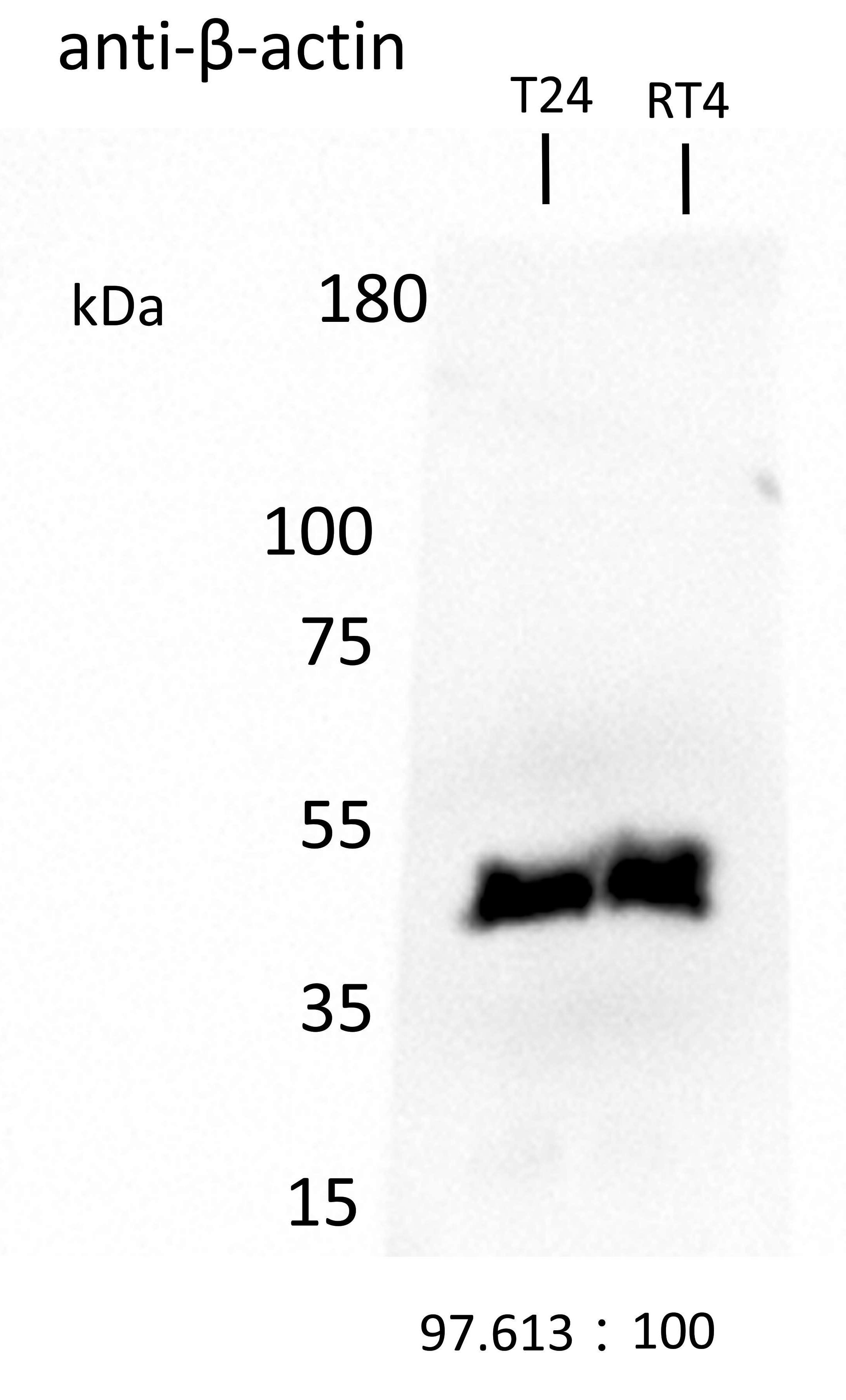

Supplement: Supplementary file 1 [file cancers-15-02487-s001.zip › Figure S3_a┬-actin.jpg]

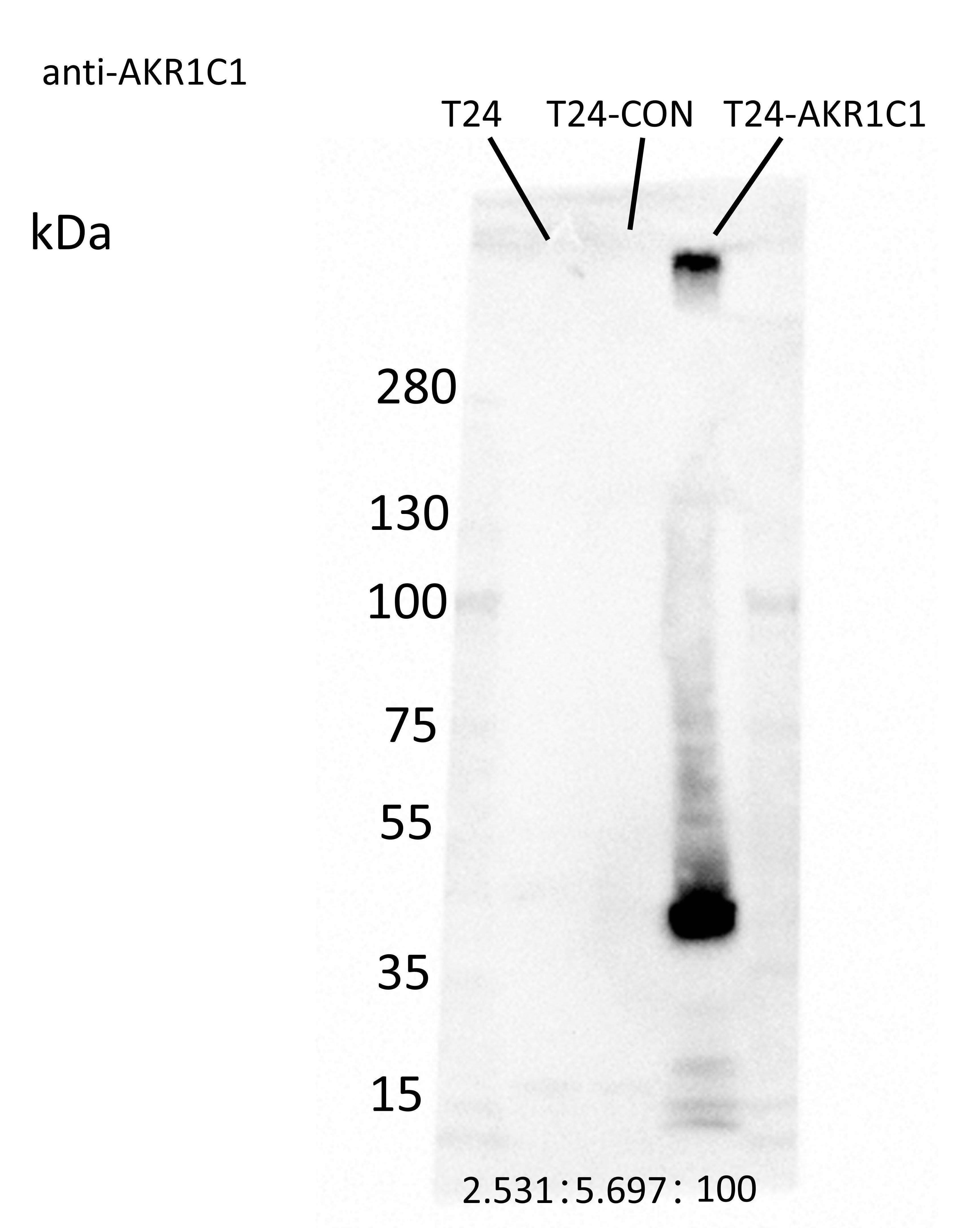

Supplement: Supplementary file 1 [file cancers-15-02487-s001.zip › Figure S4_AKR1C1.jpg]

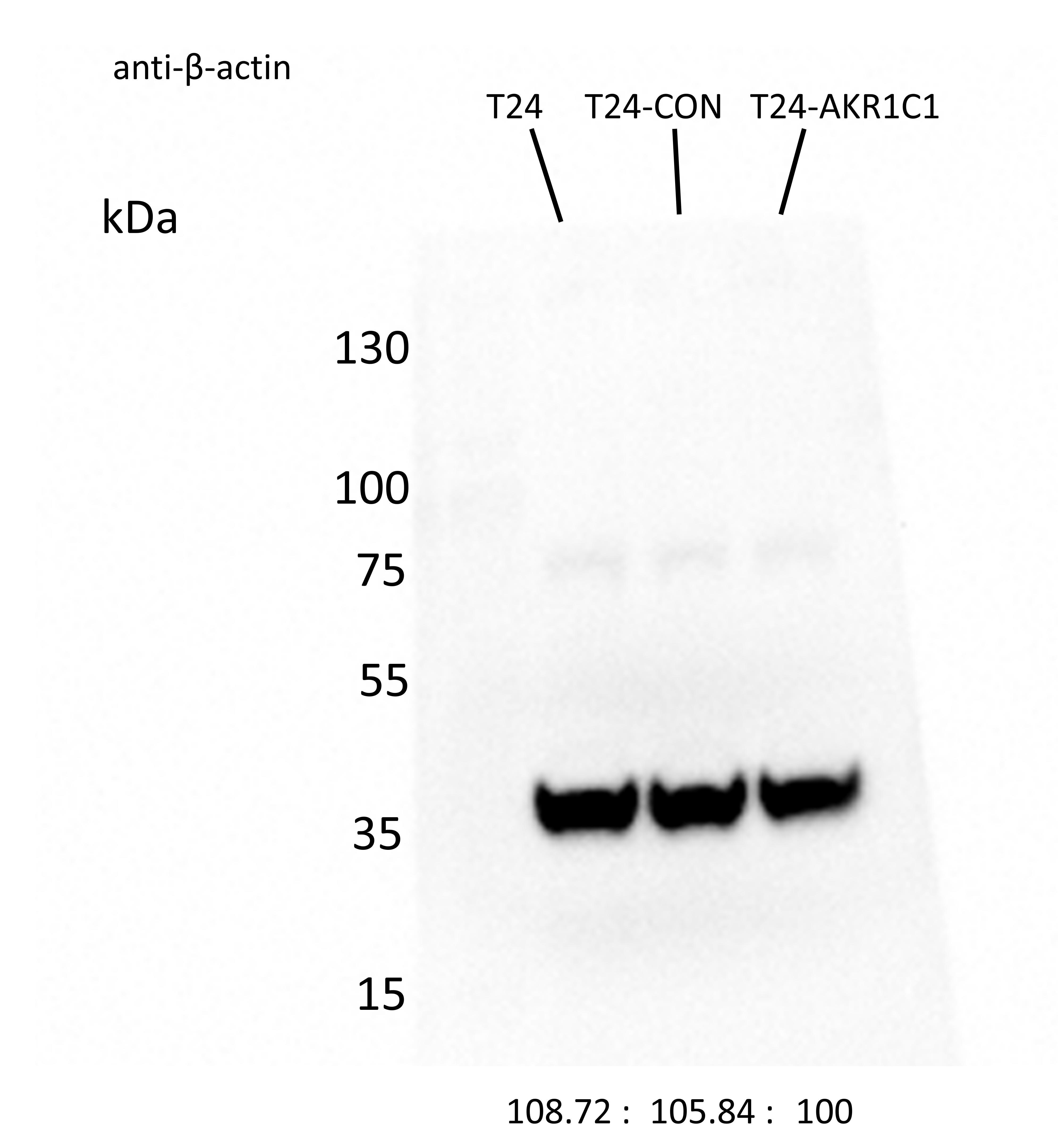

Supplement: Supplementary file 1 [file cancers-15-02487-s001.zip › Figure S5_a┬-actin.jpg]

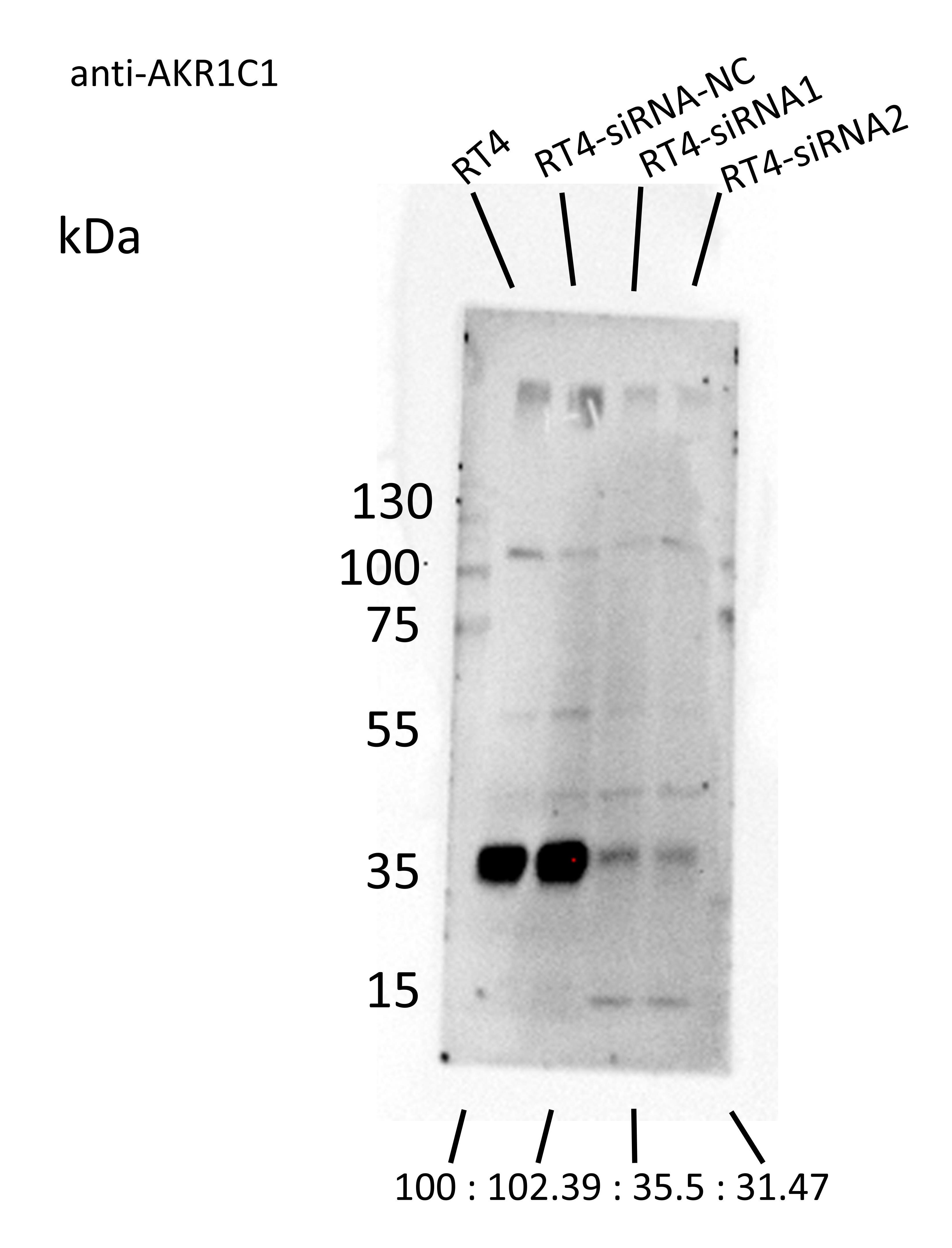

Supplement: Supplementary file 1 [file cancers-15-02487-s001.zip › Figure S6_AKR1C1.jpg]

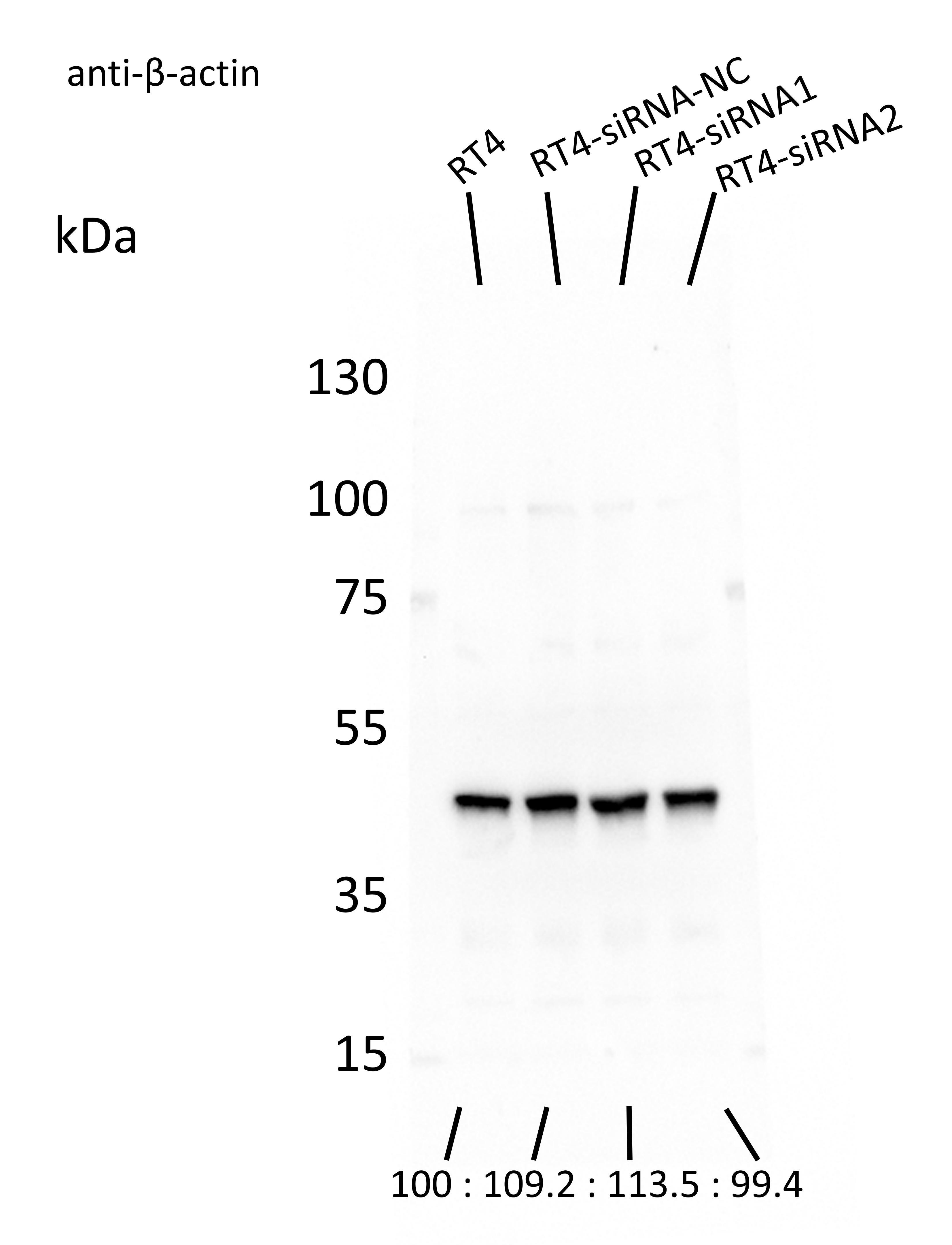

Supplement: Supplementary file 1 [file cancers-15-02487-s001.zip › Figure S7_a┬-actin.jpg]

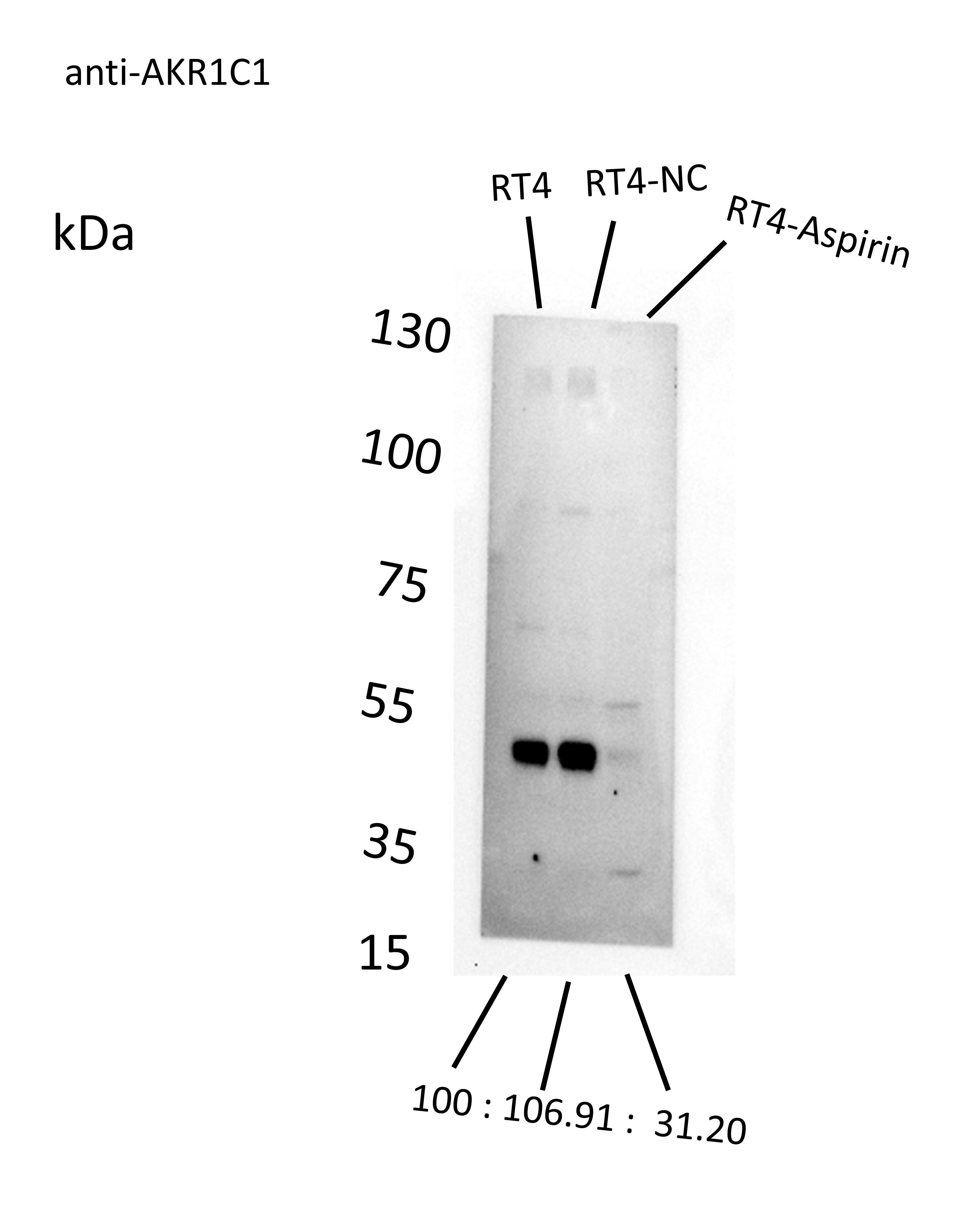

Supplement: Supplementary file 1 [file cancers-15-02487-s001.zip › Figure S8_AKR1C1.jpg]

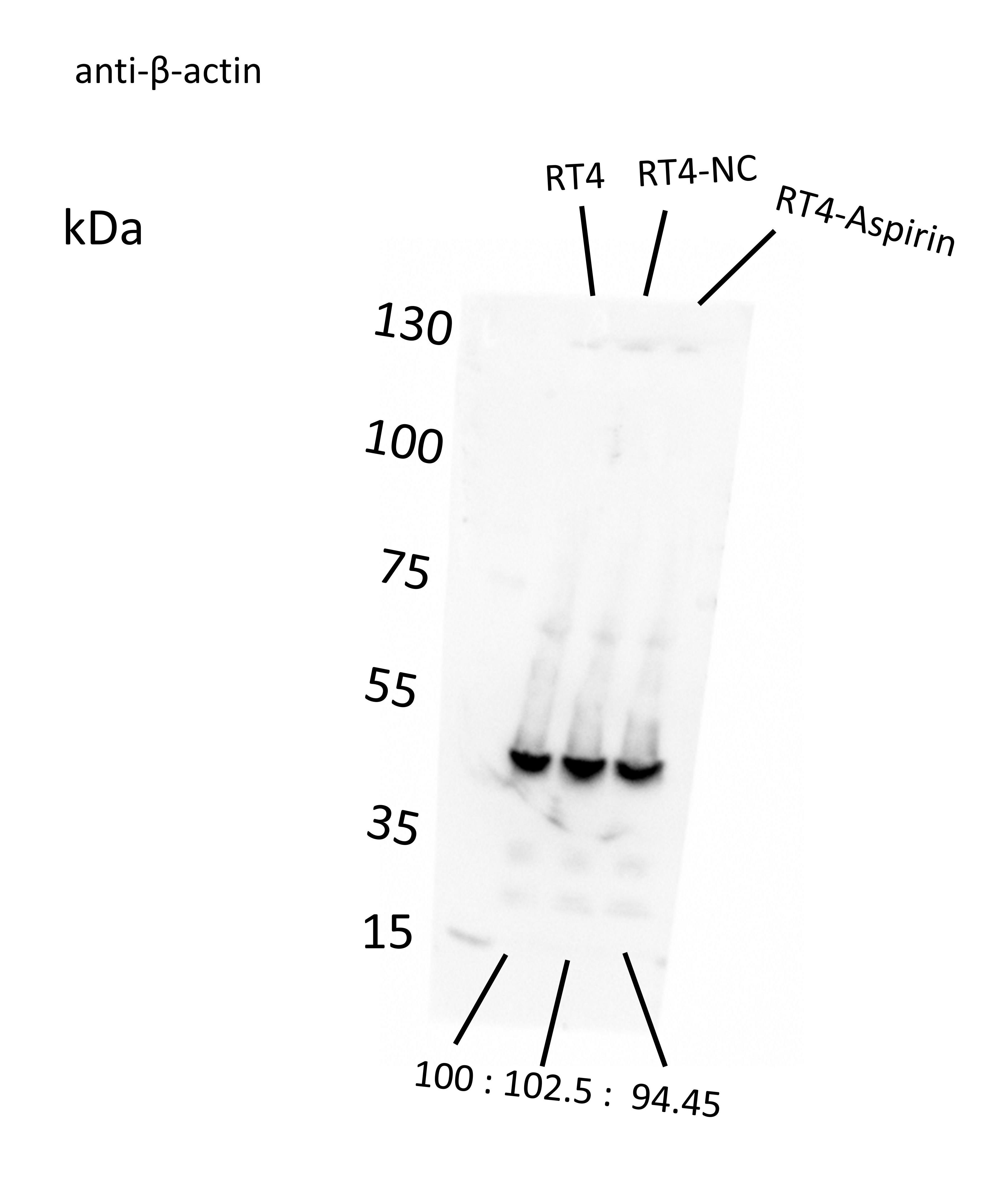

Supplement: Supplementary file 1 [file cancers-15-02487-s001.zip › Figure S9_a┬-actin.jpg]
